# Balancing Selection at the Tomato *RCR3* Guardee Gene Family Maintains Variation in Strength of Pathogen Defense

**DOI:** 10.1371/journal.pgen.1002813

**Published:** 2012-07-19

**Authors:** Anja C. Hörger, Muhammad Ilyas, Wolfgang Stephan, Aurélien Tellier, Renier A. L. van der Hoorn, Laura E. Rose

**Affiliations:** 1 Section of Evolutionary Biology, Department of Biology II, University of Munich (LMU), Planegg-Martinsried, Germany; 2 Plant Chemetics Lab, Chemical Genomics Centre of the Max Planck Society, Max Planck Institute for Plant Breeding Research, Cologne, Germany; 3 Institute of Population Genetics, Heinrich Heine University Düsseldorf, Düsseldorf, Germany; University of Georgia, United States of America

## Abstract

Coevolution between hosts and pathogens is thought to occur between interacting molecules of both species. This results in the maintenance of genetic diversity at pathogen antigens (or so-called effectors) and host resistance genes such as the major histocompatibility complex (MHC) in mammals or resistance (*R*) genes in plants. In plant–pathogen interactions, the current paradigm posits that a specific defense response is activated upon recognition of pathogen effectors via interaction with their corresponding R proteins. According to the “Guard-Hypothesis,” R proteins (the “guards”) can sense modification of target molecules in the host (the “guardees”) by pathogen effectors and subsequently trigger the defense response. Multiple studies have reported high genetic diversity at *R* genes maintained by balancing selection. In contrast, little is known about the evolutionary mechanisms shaping the guardee, which may be subject to contrasting evolutionary forces. Here we show that the evolution of the guardee *RCR3* is characterized by gene duplication, frequent gene conversion, and balancing selection in the wild tomato species *Solanum peruvianum*. Investigating the functional characteristics of 54 natural variants through *in vitro* and *in planta* assays, we detected differences in recognition of the pathogen effector through interaction with the guardee, as well as substantial variation in the strength of the defense response. This variation is maintained by balancing selection at each copy of the *RCR3* gene. Our analyses pinpoint three amino acid polymorphisms with key functional consequences for the coevolution between the guardee (*RCR3*) and its guard (*Cf-2*). We conclude that, in addition to coevolution at the “guardee-effector” interface for pathogen recognition, natural selection acts on the “guard-guardee” interface. Guardee evolution may be governed by a counterbalance between improved activation in the presence and prevention of auto-immune responses in the absence of the corresponding pathogen.

## Author Summary

Pathogens have a negative impact on the fitness of their hosts and are responsible for drastic epidemics in humans, animals, and plants. In plants, it has been thought that natural selection acts predominantly on so-called “resistance genes,” which recognize pathogens following a key-lock interaction. In this study, we demonstrate that the arms race between hosts and pathogens extends to other components of the immune system. We discovered a signature of balancing selection at the tomato *RCR3* gene, which serves as a target for pathogen-derived molecules and facilitates recognition of the pathogen via interaction with a tomato resistance gene. Functional assays of 54 *RCR3* alleles reveal that the polymorphisms underlying the observed pattern of balancing selection do not play a role in pathogen recognition, but are responsible for fine tuning the defense response of infected cells upon pathogen recognition. Therefore, the optimal *RCR3* allele depends upon a delicate balance between sufficient activation in the presence of, but avoidance of auto-activation in the absence of, the pathogen. The optimization of defense activation is likely a very important aspect of immune system evolution, especially when the selection pressure by the pathogen is variable in time and space.

## Introduction

The coevolutionary arms race between hosts and pathogens is often described as a recurrent struggle for increased resistance in hosts and evasion of recognition by pathogens [1]–[3]. The coevolutionary dynamics can be driven by negative frequency-dependent selection, leading to the maintenance of allelic diversity at genes involved in interactions between hosts and pathogens [4]–[7]. In plants, the molecular perception of pathogens and activation of defense are well understood (reviewed in [8]–[10]) and provide an ideal means to study coevolutionary processes. For interactions of plants with biotrophic pathogens, two layers of pathogen recognition and defense are commonly described: 1) the basal defense is initiated following recognition of common pathogen-associated molecular patterns (PAMPs), such as bacterial flagellin or LPS (reviewed in [11]), and 2) a specific defense response that is activated upon pathogen recognition of host-specific pathogens via gene-for-gene interactions of pathogen effectors with their corresponding resistance (R) proteins [2], [9], [12], [13]. The specific defense response typically involves a localized cell death response, called the hypersensitive response (HR), which stops the course of infection [10], [14].

The latter specific interaction between effector and R protein can be direct or indirect. Direct interactions between pathogen effectors and R proteins have been demonstrated in remarkably few cases, for example between Pita and AvrPita in the rice-*Magnaporthe* pathosystem [15] and between L and AvrL567 proteins in the flax-flax rust pathosystem [16]. However, the majority of interactions appear to be indirect, following the ‘Guard-Hypothesis’ [17], [18]. In this scenario, the pathogen effector is recognized through detection of its activity in the host. Specific target molecules in the host plant, the ‘guardees’, are modified by the activity of secreted pathogen effector molecules. This modification is detected by the R protein, which serves as the so-called ‘guard’, triggering downstream defense responses including HR.

Due to the complex interaction between the guardee, its guard and the pathogen effector, the guardee is expected to be subject to contrasting evolutionary forces [19], [20]. For example if pathogen pressure is high, positive selection on the effector-guardee interface could act to improve the detection of the effector in presence of the guard, or curtail damage caused by the effector. Alternatively, positive selection on the guard-guardee interface may improve pathogen triggered activation and/or prevent auto-activation of the defense response resulting in auto-immune response [21]. Balancing selection may act on the guardee-effector interface (due to frequency-dependent selection for pathogen recognition [22]) or guard-guardee interface (due to selection for defense activation [22], [23]), if pathogen pressure or the allele frequency of the corresponding effector vary in time or space. Although it has been shown that guardees exhibit high inter- and intraspecific diversity [20], [24], it is still unknown which evolutionary forces shape their genetic diversity and genomic structure.

To decipher the role of the guardee in the evolution of the plant immune system, we quantified the natural genetic variation and investigated the functional consequences of this variation at RCR3, a secreted papain-like cysteine protease, which is thought to be guarded by the R protein Cf-2 in tomato. Cf-2 confers resistance to the leaf mold pathogen *Cladosporium fulvum* through recognition of the fungal protease inhibitor AVR2, which physically interacts with and inhibits RCR3 ([25], reviewed in [26]). The AVR2-RCR3 interaction is thought to cause conformational changes in RCR3, which are detected by the Cf-2 protein, leading to activation of the Cf-2-mediated defense response [25], which typically involves HR.

*C. fulvum* is a host specific pathogen of the tomato clade [27]. Tomatoes (*Solanum* section *Lycopersicon*) form a monophyletic clade within the Solanaceae family. The section *Lycopersicon* includes a total of 13 species representing all described wild tomato species and the cultivated tomato *S. lycopersicum*, which diverged within the last 6 million years [28]. The native geographical distribution of wild tomato species ranges from Ecuador to northern Chile and these species are found across a range of diverse habitats including temperate deserts, Andean highlands and tropical rainforests in the Amazon basin [29]. Each species displays a characteristic geographical distribution pattern, which is defined by its habitat preference [30]. Hence, wild tomatoes are suitable model organisms to study adaptation to biotic and abiotic stress. Within the tomato clade, the obligate outcrossing species *Solanum peruvianum* exhibits the highest level of morphological and genetic diversity and has the largest and most variable habitat range including both arid and mesic habitats. Since this species harbors the greatest variation of all species in the clade of *Lycopersicon*, it is an ideal starting point to understand the interplay of functional diversity and natural selection. *S. peruvianum* diverged from its closest relatives at least 500,000 years ago [31], [32]. Adaptation to biotic factors plays an important role in evolution of this species [30], [33]–[35]. Furthermore, since the habitat range of this plant species is large and infection and transmission of pathogenic fungi such as *C. fulvum* are likely affected by climatic conditions, pathogen pressure may be variable in time and space.

Even though documentation of *C. fulvum* in wild populations of tomato is lacking, empirical studies suggest that this fungus is a natural, coevolving pathogen of wild tomato species. Wild tomato species (*S. peruvianum*, *S. pimpinellifolium* and *S. habrochaites*) can be infected by *C. fulvum* and respond with different levels of resistance and susceptibility to pathogen challenge [36]. The observed differences vary within and between these three species suggesting variability in historical pathogen pressure. Furthermore, resistance genes to *C. fulvum* are present and functional in these wild tomato species [37] and have been introgressed from resistant accessions into the cultivated tomato [38]. A previous study reported high diversity at the *RCR3* gene among different (wild) tomato species and suggested that the elevated amino acid variation at this locus might translate into functional diversity upon pathogen challenge [24].

Here we describe the natural variation occurring at the *RCR3* locus in several wild tomato species with particular focus on a set of individuals originating from a population of the species *S. peruvianum*. Previous studies of other resistance genes in this species indicate that pathogen pressure is a significant evolutionary force, at least in some parts of the species range [33]–[35]. Moreover, high levels of polymorphism in this species provide sufficient power for population genetic analyses. In fact, we show that nucleotide and amino acid diversity at the *RCR3* locus present in the Tarapaca population of *S. peruvianum* reflect the total diversity observed in interspecific comparisons across the whole tomato clade [24].

Combining a population genetic with a four-pronged functional approach, we show that the evolutionary history of the *RCR3* locus is characterized by balancing selection, recent gene duplication and frequent gene conversion in *S. peruvianum*. The *RCR3* gene forms a young gene family in this species and a closely related sister species. Two differentiated sequence types are maintained within and across *RCR3* loci. In contrast to other studies that find variation in pathogen recognition segregating at resistance loci [33], [39], we find evidence for variation in the activation of the defense response. Our results suggest that coevolution between the guardee and its guard rather than with the pathogen effector is the major force in the evolution of the *RCR3* locus.

## Results/Discussion

### The *RCR3* locus is duplicated in *S. peruvianum* and its sister species

**Figure 1:**
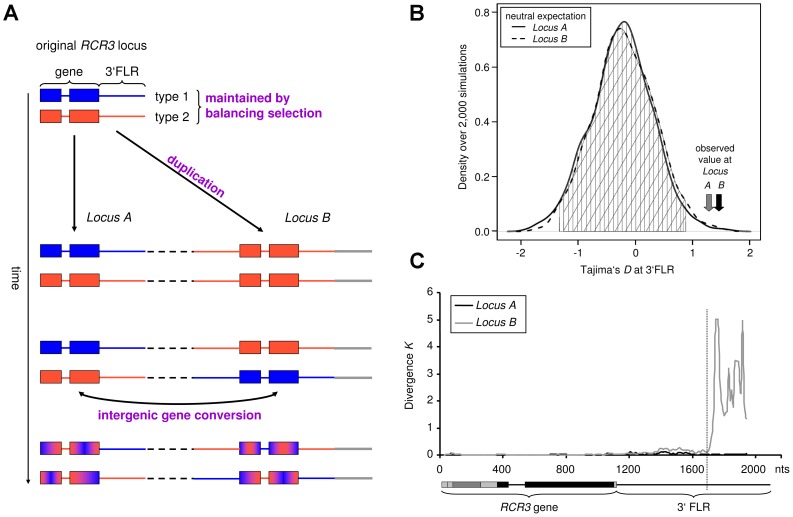
Evolutionary history of the *RCR3* gene family (*Locus A* and *B*). (A) Proposed scenario of *RCR3* gene family evolution. The duplication event introduced one of the sequence types (here the red type) into a new genomic location (*Locus B*, indicated by grey line). Subsequently, the second sequence type was exported from the original *RCR3* locus (*Locus A*) to *Locus B*. Frequent gene conversion between the two duplicates homogenizes the ORFs, but not the 3′FLRs. (B) Distribution of neutral expectation of *D*_T_ in 3′FLRs based on 2,000 coalescent simulations. The grey vertical line indicates the observed average at the reference loci. The observed values at the *RCR3* FLRs are outside the 99% confidence intervals (indicated by vertical and diagonal lines). (C) Divergence between *Locus A* and *Locus B* to *S. lycopersicum RCR3*. The structure underneath the x-axis represents the gene and the 3′FLR. The vertical dashed line approximately 580 bp downstream of the stop codon indicates the likely insertion point of the duplicated *RCR3* segment into a novel genomic location.

To investigate the evolutionary history of the *RCR3* locus, we cloned and sequenced *RCR3* alleles from 28 individuals of multiple wild tomato species (*S. chilense*, *S. chmielewskii*, *S. corneliomulleri*, *S. habrochaites*, *S. lycopersicoides*, *S. pennellii*, *S. peruvianum* and *S. pimpinellifolium*) and the cultivated tomato *S. lycopersicum* (Table S2). This approach revealed that *RCR3* forms a gene family with at least two paralogs in *S. peruvianum* and its sister species *S. corneliomulleri*. These paralogs are more closely related to *RCR3* than to other cysteine proteases, including *PIP1*, which cluster in the same genomic region [40]. The duplication of the *RCR3* locus appears to be restricted to *S. peruvianum* and *S. corneliomulleri*, since no evidence for a duplication event was found in the draft genome of the cultivated tomato or in the other tomato species investigated in this study. However, we cannot exclude the existence of more diverged paralogs in the other tomato species studied, which may not have been detected through our sequencing approach. The *RCR3* paralogs detected in this study could not be unambiguously distinguished from one another based on sequence divergence in the *RCR3* open reading frame (ORF). Therefore, we cloned and sequenced the flanking regions (FLRs) of 43 alleles from one *S. peruvianum* population (LA2744 from Tarapaca, Chile, described also in [34], [35]) and defined their genomic origin relative to the cultivated tomato through BLAST and phylogenetic analyses. These analyses showed consistent results: FLRs that corresponded to the orthologous *RCR3* containing region of the cultivated tomato based on significant BLAST hits clustered together in the phylogenetic tree. FLRs that mapped to other genomic locations in *S. lycopersicum* formed distinct clusters (Figure S1). The analyses of the *RCR3* FLRs revealed that the *RCR3* gene was duplicated at least twice in *S. peruvianum* – the duplicates are named *Locus A*, *Locus B* and *Locus C* hereafter. All 5′ flanking regions matched the *RCR3* locus from *S. lycopersicum* over the full sequenced length reaching 400 to 900 bp upstream of the gene. This indicates that the duplicated region extends further upstream of the *RCR3* gene. In contrast, based on BLAST hits, only a portion of the 3′FLRs matched the *RCR3* locus from *S. lycopersicum*. At approximately 580 bp downstream of the stop codon, *Locus B* diverges from both *Locus A* and the *S. lycopersicum* sequence (Figure 1C). This marks the likely insertion point of the duplicated *RCR3* segment into a novel genomic location at the time of origin of this new duplicate. BLAST hits for the 3′FLR of *Locus B* alleles beyond this breakpoint mapped to a genomic region located approximately 8.2 kb downstream of the *RCR3* locus in the tomato genome. *Locus C* is characterized by a large deletion in the 3′FLR relative to the *S. lycopersicum* sequence and it was not possible to map it using the draft tomato genome. The phylogenetic and BLAST analyses of the flanking sequences indicated that alleles from *Locus A* have the highest sequence similarity to *RCR3* from other *Solanum* species (*S. lycopersicum* and *S. pimpinellifolium*) and sequence divergence lies within the range of the overall sequence divergence observed between *S. lycopersicum* and *S. peruvianum* (which ranges from 0.0039 to 0.0589 across 50 loci, [41]). It is therefore likely that alleles from *Locus A* are orthologous to the *RCR3* gene in the other species, in which the *RCR3* gene is not duplicated. This implies that *Locus B* and *Locus C* are more recently derived duplicates of *Locus A* in *S. peruvianum*.

Our approach allowed us to unambiguously match 27 *RCR3* sequences with their corresponding 3′FLR and therefore assign 27 of 43 *RCR3* sequences to the different loci: 14 alleles to *Locus A*, nine alleles to *Locus B* and four to *Locus C* (Table S2). The copy number of the gene varies between individuals of *S. peruvianum* and no individual seemed to carry all three *RCR3* copies. However, all but two tested individuals carried alleles that were assigned to two different *RCR3* loci and, in most cases, at least one allele originated from *Locus A* (Figure S1). For population genetic analyses, only alleles that could be unambiguously assigned to their corresponding locus were used. Due to small sample size (*n* = 4), alleles originating from *Locus C* were excluded from the analysis. The genomic origin of each assigned allele is indicated by the respective letter (A, B or C) in the nomenclature used in this study.

### The two *RCR3* loci undergo frequent gene conversion

Gene duplication and subsequent (functional) divergence of duplicates are typical mechanisms generating diversity at genes involved in host-pathogen coevolution [42]–[44]. However, young duplicates that have not had time to diverge from one another can be homogenized by frequent intergenic gene conversion [42]. The high sequence similarity between the *RCR3* ORFs and the presence of copy number variants within populations are consistent with the recent origin of the *RCR3* gene family in *S. peruvianum*. We therefore developed an Approximate Bayesian Computation (ABC) method [32], [45] to evaluate whether gene conversion occurs and, if so, at what rate [46]. A model of evolution with gene conversion was largely favored over a model without gene conversion (Bayes Factor>1,000). The population gene conversion rate *C* between the *RCR3* ORFs was consistently estimated to be significantly greater than zero (*C* = 1.08, credibility interval CI = [0.19–7.7], Figure S2, Table S3) and more than 100 times larger than the population mutation rate estimated at 14 reference loci in *S. peruvianum* (0.014; Table S4) or at the *RCR3* gene (0.0085; Table S4).

A survey of the site frequency spectrum (SFS) of shared and private polymorphisms [47] also confirms this high rate of gene conversion (Text S1, Figure S3). We therefore suggest that functional divergence between the two copies on the protein level is unlikely at this stage of evolution because adaptive mutations appearing at one locus can be transferred to the other locus by gene conversion [46]. In contrast, signatures of gene conversion could not be detected at the 3′FLRs based on ABC analysis and the shape of the SFS (fewer shared polymorphisms, excess of fixed differences between loci, Text S2, Figure S3). This suggests that gene conversion does not happen as frequently in the 3′FLRs of the *RCR3* gene as compared to the *RCR3* ORFs.

### Two differentiated sequence types are maintained in the *RCR3* gene family by balancing selection

**Figure 2:**
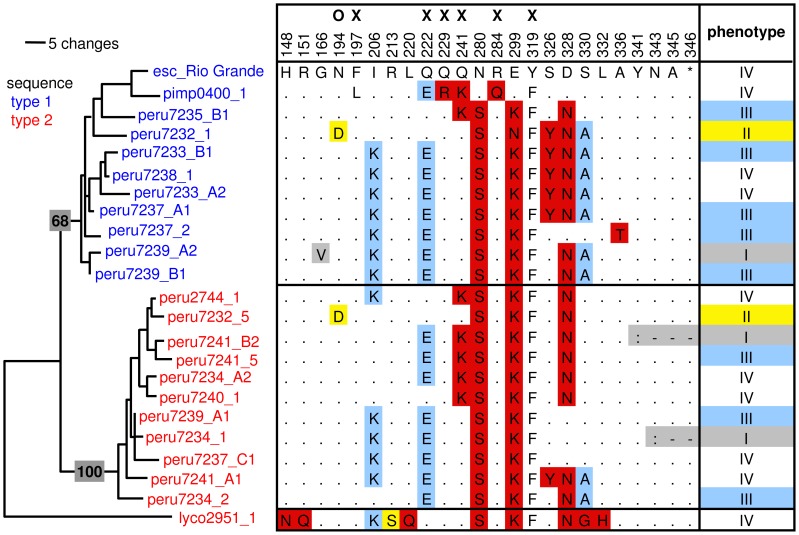
Overview of a subset of RCR3 protease domain haplotypes, their phenotype, and position in the *RCR3* gene tree. The tree (MP) containing a subset of *RCR3* sequences is based on Figure S4 with *S. lycopersicoides* as outgroup. Bootstrap support is indicated on the branches. The protein sequence of *S. lycopersicum* is used as a reference (esc_Rio Grande). Sequences are named according to their species, their accession or individual number and their origin from *Locus A*, *B* or *C* in those cases for which unambiguous assignment was possible. Sequences from sequence type 1 are labeled in blue; sequences from sequence type 2 are labeled in red. In the haplotype matrix, identical amino acids are indicated with dots, similar amino acids with the according letter, dissimilar amino acids with red and functionally relevant amino acid changes with yellow and blue according to their phenotypic association using all RCR3 constructs. X = mutation putatively causing incompatibility with Cf-2 [56], O = mutation causing insensitivity to inhibition by AVR2 [24]. Phenotype I = not accumulated in AF, II = not inhibited by AVR2 and no HR, III = inhibited by AVR2 and attenuated HR, IV = inhibited by AVR2 and strong HR.

We analyzed sequence variation within the population to evaluate which selective forces act on the *RCR3* gene. Phylogenetic analyses of the coding sequence of all assigned *RCR3* alleles revealed two differentiated sequence types (Figure 2 and Figure S4), which segregate within all three loci. The haplotypic structure of the sequence types is mainly due to two different intronic sequence types and variation in linkage disequilibrium with this intron. The two sequence types are highly differentiated from one another: The index of fixation at *RCR3* (*F*_ST_ = 0.311) is higher than the average *F*_ST_ between populations of *S. peruvianum* at eight reference genes (*F*_ST_ = 0.198, minimum 0.081, maximum 0.352, [48]). However, polymorphism within each sequence type is low (π_sequence type 1_ = 0.007, π_sequence type 2_ = 0.005) consistent with the maintenance of the two sequence types via long-term balancing selection.

To evaluate whether natural selection contributed to the maintenance of the distinct sequence types at the *RCR3* locus, several population genetic statistics were calculated for the alleles of *RCR3 Locus A* and *Locus B*. Putative pseudogenes (see below and Text S3) were excluded from these analyses. To rule out demographic effects, which could interfere with the detection of the signature of natural selection acting at the *RCR3* locus, all statistics were compared to a set of 14 reference loci that had previously been sequenced in the same individuals of *S. peruvianum* [49], [50].

We computed Tajima's *D* (*D*_T_), which summarizes the SFS of mutations in a given dataset [51]. Positive *D*_T_ values indicate an excess of polymorphism at intermediate frequency, a pattern indicative of balancing selection. A sliding window analysis depicting *D*_T_ across the entire *RCR3* ORFs revealed regions with highly positive values. To test whether *D*_T_ at the *RCR3* ORF would globally deviate from neutrality, we derived the expected distributions of *D*_T_ for the studied population under neutrality, taking demography and the respective gene conversion rate into account (Texts S1 and S2). The observed values at the *RCR3* ORFs do not deviate significantly from neutrality (Figure S5, Table S4). However, the 3′FLRs of both loci exhibit significantly positive *D*_T_ values compared to the expected neutral distribution for this population (Figure 1B, Text S2, Figure S6). Taken together, our findings suggest the following evolutionary scenario for the *RCR3* loci in *S. peruvianum* (Figure 1A). Since both sequence types segregate at each locus and the FLRs show positive Tajima's *D* values, the two sequence types most likely pre-date the formation of the gene family and have been maintained by balancing selection. At the initial time of duplication, only a single sequence type would have been transferred to the new genomic region (8.2 kb downstream of *Locus A* in the *S. lycopersicum* genome), for example sequence type 1 from *Locus A* to *Locus B*. Then, following the origin of *Locus B*, the second sequence type (*e.g.* type 2) was also introduced at *Locus B* by recombination events such as gene conversion. High levels of recombination within the coding region (perhaps via gene conversion as described above) have subsequently intermixed the two sequence types and likely obscured the signature of balancing selection in the coding region by whittling down the region targeted by natural selection. In contrast, the signature of balancing selection is apparent in the 3′FLRs, where gene conversion does not occur as frequently.

### The balanced polymorphism underlies differences in the strength of HR

The presence of two distinct sequence types differentiated especially in their intron sequences suggests three potential targets of selection: 1) selection on different regulatory motifs in the intron, 2) selection for different splicing variants or 3) selection on one or more amino acid polymorphism(s) in linkage with the intron. *In silico* analysis did not reveal different regulatory motifs between the two intronic sequence types, although we cannot rule out the possibility that novel regulatory motifs have been overlooked. Nucleotide sequencing of mRNA from the two sequence types did not indicate the existence of different splicing variants at the *RCR3* locus. Therefore, we reason that balancing selection is most likely acting on amino acid polymorphism(s) linked to the intron.

To evaluate functional differences between sequence types at the protein level, we took a four-pronged approach. Using an over-expression vector *in planta*, we first evaluated whether protein accumulated for all sequence types in apoplastic fluids (AFs) of *Nicotiana benthamiana*. In total, 54 different allelic protein variants were chosen for these assays as follows. Eleven of these protein variants were chosen from the set of 27 alleles of *S. peruvianum* that could be assigned to *Locus A*, *B* or *C*. These eleven variants represented the protein diversity found in this set of *S. peruvianum* alleles. These alleles originated from all three loci and included both sequence types. The remaining 43 variants were chosen from the set of *S. peruvianum* alleles that could not be assigned to their corresponding locus and from closely related tomato species to maximize the amount of amino acid variation assayed. Of the total number of tested alleles, 47 were detected in AFs by Western blotting (Figure S7, Table S5). The remaining seven *RCR3* proteins did not accumulate in independent expression assays, although the accumulation of mRNA was confirmed by RT-PCR (class I alleles in Figure 2, Figure S8). One of these alleles originated from *S. corneliomulleri* and six alleles originated from *S. peruvianum* (one from *Locus A*, one from *Locus B*, and four could not be assigned to their corresponding locus). In all cases in which no protein accumulated, the causative mutations (frame shifts leading to premature stop codons in five of these alleles and point mutations in the remaining two) could be identified (Text S3, Figure S9, Table S5). Since these seven alleles appear to be pseudogenes, they were excluded from population genetic analyses described above.

The second assay was a protease enzymatic assay. The activity of the RCR3 proteins in AFs was detected by Activity-based Protein Profiling (ABPP) using fluorescent DCG-04. DCG-04 is an inhibitor of papain-like cysteine proteases and reacts irreversibly and covalently to the active site cysteine of proteases in an activity-dependent manner [52]. This assay has been applied frequently to detect the activity of plant proteases and their inhibition by pathogenic protease inhibitors [24], [25], [53]–[55]. All 47 expressed RCR3 proteins could be labeled by DCG-04 to similar levels, confirming that they all are active proteases (Figure S10, Table S5).

Our third and fourth functional assays were designed to detect differences among alleles in their sensitivity to AVR2 and in their ability to elicit HR upon co-infiltration with and without AVR2 into *rcr3*-mutant tomato plants (*cv.* Money Maker Cf-2/rcr3-3, [56]). Inhibition assays based on competitive ABPP were performed to determine which RCR3 can be inhibited by the fungal protease inhibitor AVR2. Of the 47 tested RCR3 proteins, 41 (including all tested alleles from *Locus A*, *B* and *C*) were inhibited by AVR2 resulting in the activation of the Cf-2 dependent defense response *in planta* (alleles in classes III and IV in Figure 2, Figure S11, Table S5). The six alleles that failed to be inhibited by AVR2 were isolated from individuals of *S. peruvianum* and *S. chilense* (alleles in class II in Figure 2). A single nonsynonymous substitution at position 692, resulting in a change from asparagine (N) to aspartic acid (D) at position 194 in the protein (N194D), is significantly associated with this phenotypic difference (at 1% after Bonferroni correction; *R* ^2^ = 0.842, *P* = 1.05×10^−26^, Figure S12). This supports previous results using site directed mutagenesis by Shabab *et al.* (2008) [24], which found that RCR3 alleles carrying the N194D substitution are insensitive to inhibition by AVR2. Additionally we show here that alleles that carry the N194D mutation fail to activate the defense response *in planta*, in the presence of AVR2 (Figures S11 and S12). Due to the large sample size used in this study (54 alleles), we had power to detect epistatic interactions between amino acid variants, such as the substitution R151Q in an allele carrying the N194D mutation (peru1954_1). This allele with the Q variant at site 151 was inhibited by AVR2, contrary to other alleles with the D variant at site 194, implicating potential epistatic interactions between these two polymorphisms (Figures S9 and S13, Table S5). Among all tested alleles that do not carry the N194D substitution only a single allele, peru7233_2, did not induce HR despite being sensitive to inhibition by AVR2 (Table S5). This allele has one amino acid difference (R138I) compared to other alleles that induced HR upon co-infiltration with AVR2 (Figure S9).

In addition to the N194D polymorphism, nucleotide polymorphisms at positions 717 (synonymous mutation) and 750 (causes amino acid difference R213S) were associated with insensitivity to inhibition by AVR2 (Figure S12, *R* ^2^ = 0.254, *P* = 2.9×10^−6^; and *R* ^2^ = 0.336, *P* = 9.8×10^−8^). However, since alleles that have the polymorphism at bp 750 (R213S), but not N194D, can be inhibited by AVR2 and elicit HR *in planta*, it is likely that the association between phenotype and sequence variation for this polymorphism is due to linkage disequilibrium. In our data set encompassing nine *Solanum* species, the amino acid substitution N194D was found exclusively in six individuals of *S. peruvianum* and *S. chilense* and was only represented by two alleles in the dataset used for the population genetic study. Therefore, it is unlikely that this polymorphism alone can account for the signature of balancing selection at this locus.

**Figure 3:**
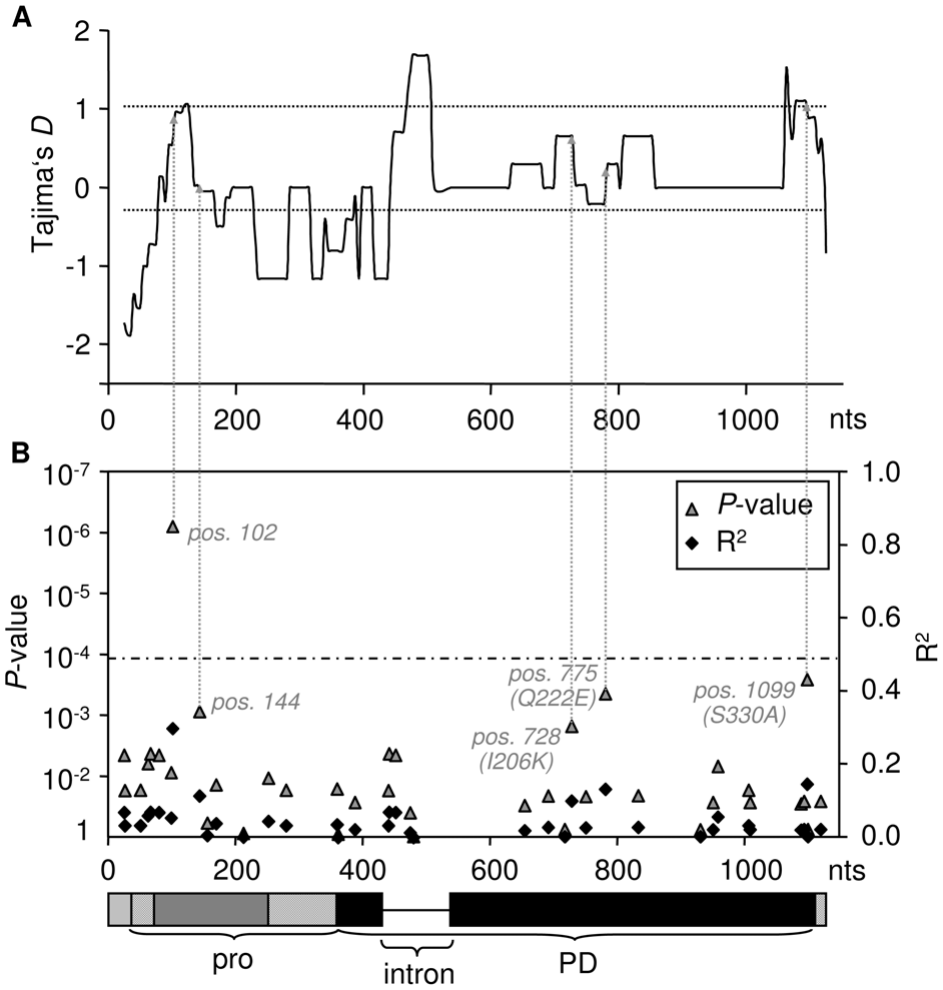
Association of the weak HR phenotype with sequence polymorphism. (A) Tajima's *D* across the two *RCR3* loci. The dotted lines indicate the mean and maximum of *D*_T_ measured at 14 reference loci. *D*_T_ is elevated at sites that are associated with the weak HR compared to the neutral expectation ( = 0) and even more so compared to the genomic average. (B) Association of SNPs and phenotypic variation in HR response with *P*-value of the correlation (left y-axis) and correlation coefficient (right y-axis). Values were corrected using the Bonferroni-method (dashed line indicates the 5% significance threshold). The dashed arrows indicate the corresponding Tajima's *D* values for all polymorphisms associated with the attenuated HR phenotype. The structure of the gene is indicated below the x-axis (grey and black boxes are the exons, pro = pro-domain, PD = protease domain).

According to the ‘Guard-Hypothesis’ the defense response relies upon two distinct events: modification of the guardee by the effector and activation of the defense signaling through the guard molecule [17], [18]. Our approach enabled us to investigate both events. The inhibition assays did not show differential sensitivity for modification by AVR2 among most of the tested RCR3 alleles besides the alleles carrying the N194D mutation. However, our functional assay for HR activation revealed differences in the strength of activation of the defense response. Despite similar levels of sensitivity to AVR2, the tested RCR3 variants differ substantially in the strength of the defense response they elicit, with 13 protein variants (two of which originating from *Locus A* and two originating from *Locus B*) showing weaker HR (slower HR, smaller extent of cell death) compared to the others (alleles in class III in Figure 2, Figure 3, and Figure S11, Table S5). Five SNPs are correlated with phenotypic variation in the strength of the HR; one (at nucleotide position 102) remaining statistically significant at the 1% level after Bonferroni correction (Figure 3, Figures S9 and S13). All five mutations associated with this phenotype are linked with one another and with the intron despite frequent gene conversion at the locus. The most likely polymorphisms targeted by natural selection are at nucleotide positions 728 (*R* ^2^ = 0.1, *P* = 0.015), 775 (*R* ^2^ = 0.132, *P* = 0.0044) and 1099 (*R* ^2^ = 0.146, *P* = 0.0026) since all three polymorphisms result in amino acid changes (I206K, Q222E and S330A). One of these polymorphisms (Q222E) is identical to an amino acid substitution that has previously been suggested to be involved in auto-necrosis due to incompatibility between RCR3 and Cf-2 ([56], Figure S13). In a putative structural model of the RCR3 protease domain the remaining two amino acid polymorphisms are located on the same protein surface as four additional positions that may be involved in the incompatibility between RCR3 and Cf-2 ([56], Figure S13). All three amino acid changes result in dissimilar amino acid substitutions and could have an impact on the protein conformation and function, while the two polymorphisms at synonymous sites (Figure 3B) may affect *RCR3* transcript stability and could also be targets of selection [57]. Together with the intron, all five mutations are located in the regions of positive Tajima's *D* values in the sliding window analysis and likely underlie the signature of balancing selection at the *RCR3* locus (Figure 3).

### A balanced polymorphism underlying coevolution between guard and guardee?

Previous studies on *R* gene evolution demonstrated the maintenance of variation in pathogen recognition via balancing selection [4], [5], [34]. Our combination of functional assays, population genetic tools, computational and statistical methods allowed us to pinpoint specific amino acid polymorphisms affecting guardee function. We find that a balanced polymorphism is present at each copy of the young guardee gene family in *S. peruvianum* because (or in spite) of the homogenizing force of gene conversion. Balancing selection, gene duplication and gene conversion are mechanisms known to play a major role in *R* gene evolution [4], [42] and appear to be important in the evolution of the guardee *RCR3*, at least in *S. peruvianum*. The signature of balancing selection persists in this species and while there is no evidence that gene duplication and gene conversion are involved in evolution of *RCR3* in other species, it is possible that balancing selection could play a role in the evolution of *RCR3* also in other species. However, contrary to what is reported at *R* genes, variation in pathogen recognition does not seem to underlie the balanced polymorphism at *RCR3*. Instead, our results suggest that variation in the activation of the defense response, rather than effector recognition *per se*, underlies the balanced polymorphism. Two alternative scenarios of the evolution of *RCR3* could explain these observations:

1) The diversity detected at the *RCR3* locus could be due to coevolution with allelic types of *AVR2* or with other pathogen effectors not included in this study. In our assay, the phenotypic response to a single allelic type of AVR2 was similar in all but six tested RCR3 alleles. Therefore, functional differences between the different RCR3 alleles regarding interaction with other allelic variants of AVR2 are improbable. Furthermore, the polymorphisms underlying the signature of balancing selection were not associated with phenotypic variation in AVR2 sensitivity. Therefore, balancing selection for differential recognition of AVR2 alone cannot account for the maintenance of the two functional types at *RCR3*.

RCR3 can be targeted by other pathogen effectors, including EPIC1/2B which are secreted by the oomycete *Phytophthora infestans* [58]. These effectors are protease inhibitors and are thought to target cysteine proteases similar to RCR3 close to the substrate binding groove [59]. Unlike the N194D mutation, which has been shown to have indeed an effect on the interaction between RCR3 and AVR2, the polymorphisms underlying the signature of balancing selection are not located close to the putative site of interaction between RCR3 and these effectors. It is therefore unlikely that the observed signature of balancing selection is due to coevolution between RCR3 and other protease inhibitors. Note however, that the molecular and structural details of the interaction between RCR3 and these other effectors and its role in disease resistance are not yet well-understood. It will be of great interest to study the coevolution between *RCR3* and its full effector repertoire once their roles in disease resistance have been resolved.

2) Alternatively, variation could be maintained at the *RCR3* locus through coevolution at the interface between guardee and interacting host molecules and involve balancing selection for resistance/susceptibility at the level of defense signal activation. Balanced polymorphisms for resistance and susceptibility due to a potential cost of resistance and/or ecological/epidemiological factors have been studied theoretically [22] and empirically [4], [60] at *R* genes. To our knowledge, this study provides first evidence that this mechanism can also drive guardee evolution. In the case of the *RCR3* gene, a potential cost of resistance could be the result of coevolution with its guard *Cf-2*, which also exists as a gene family in wild tomatoes (*S. pimpinellifolium*; [61], [62]). The interaction between RCR3 and Cf-2 requires a precise matching between allelic variants. A mismatch between allelic variants from the closely related species *S. lycopersicum* (RCR3^esc^) and *S. pimpinellifolium* (RCR3^pim^) results in an auto-necrotic response [56] and may be an example of Dobzhansky-Muller incompatibility between tomato species [63]. One of the amino acid changes differing between RCR3^pim^ and RCR3^esc^ and potentially contributing to the reported incompatibility between RCR3 and Cf-2 (Q222E, [56]) is associated with the attenuated HR phenotype observed in this study. The remaining amino acid changes associated with this phenotype are not identical to, but are located on the same protein surface as the potential causative mutations of the RCR3-Cf-2 incompatibility (Figure S13). The amino acid changes underlying the balanced polymorphism at the *RCR3* locus and causing differences in the strength of HR therefore likely play a role in the interaction between RCR3 and Cf-2. Different combinations of RCR3-Cf-2 allelic variants might thus result in differential activation of the defense response.

For practical reasons, we tested the RCR3 proteins in standard *S. lycopersicum* backgrounds. Since we did not conduct our assays in *S. peruvianum*, we cannot exclude the possibility that RCR3 and Cf-2 function differently in this species. However, the fact that RCR3 alleles from *S. peruvianum* do activate resistance in the presence of Cf-2 alleles in the *S. lycopersicum* background as expected from previous studies in *S. lycopersicum* and *S. pimpinellifolium* suggests that this interaction is conserved throughout the tomato clade.

Furthermore, even if the RCR3-Cf-2 interaction is not conserved in *S. peruvianum*, *RCR3* may be coevolving with other host endogenous molecules which could explain the pattern of variation observed at *RCR3*.

Since in our study all RCR3 alleles were tested in an identical genetic background, some alleles may not be matched with their optimal Cf-2 partner, explaining the observed attenuated response for some pairings of RCR3s with Cf-2. However, we are confident that the different observed HR phenotypes are not an artifact of using a particular Cf-2 protein, because both *RCR3* types are maintained by balancing selection. In nature, while an attenuated response due to weaker interaction between guard and guardee may result in reduced resistance in the presence of the pathogen, these alleles may be selectively advantageous in the absence of the pathogen, because they carry a lower cost and/or risk for activation of auto-immunity [23]. Therefore, the optimal *RCR3* allele will depend upon this delicate balance between sufficient activation in the presence of the pathogen, but limited risk for auto-activation in the absence of the pathogen, explaining why both *RCR3* types segregate within a single population. The optimization of defense activation may be a very important component of guard-guardee coevolution, especially when the selection pressure by the pathogen is variable in time and space.

## Materials and Methods

### Plant material and DNA sequencing

For population genetic and functional analyses, the ORF of the *RCR3* gene was cloned and amplified from genomic DNA from eleven heterozygous individuals of *S. peruvianum* (accession LA2744 from Tarapaca, Chile), collected by Charles M. Rick (Table S2). Seeds from different field collected plants were grown under standard greenhouse conditions in Davis, CA. DNA was isolated using the CTAB method (Doyle and Doyle, 1987). Alleles from single individuals from eight additional species of *Solanum* were cloned and sequenced and their *RCR3* alleles were functionally tested (Table S2). These species included: *S. peruvianum* (accessions LA1954 and LA0446), *S. chilense* (accessions LA2748, LA1930 and LA1958), *S. corneliomulleri* (accessions LA1274 and LA1973), *S. pimpinellifolium* (accession LA0400), *S. lycopersicum* (cv. VFNT Cherry and cv. Rio Grande), *S. chmielewskii* (accession LA3653), *S. habrochaites* (accession LA1777), *S. pennellii* (accessions LA0716 and LA3791). For outgroup comparisons, the *RCR3* gene from *S. lycopersicoides* (accession LA2951) was sequenced and tested. All accessions were obtained from the Tomato Genetics Resource Center (TGRC) in Davis, CA. Plant growth conditions and DNA extraction for these additional accessions (with exception of LA1954, LA0446, LA2748, LA1930, LA1958, LA1274 and LA1973) were identical as for *S. peruvianum* from Tarapaca (LA2744). DNA from these other accessions was extracted using the Dneasy DNA Extraction Kit (Qiagen).

The *RCR3* gene was identified using the *RCR3* reference sequence from *S. lycopersicum* cv. ‘*Mogeor*’ (GenBank, accession number AF493234). Restriction sites for cloning were introduced into the primer sequences, which were designed to cover the start and stop codon (Table S1). The gene was PCR amplified using the Phusion proofreading polymerase (Finnzymes, Espoo, Finland) and cloned into Zero Blunt TOPO vectors (Invitrogen, Carlsbad, CA). Direct sequencing of PCR products and sequencing of miniprepped plasmid DNA from clones were conducted in parallel (Big Dye Terminator v 1.1, Applied Biosystems). Sequencing was performed according to the Sanger sequencing protocol using the DNA analyzer ABI 3730 (*Applied Biosystems & Hitachi*). Multiple clones per gene per individual were sequenced and ambiguous positions were compared to the direct sequences from the original PCR products. When necessary, independent rounds of PCRs, cloning and sequencing were conducted to resolve ambiguities. Raw sequences were edited and aligned in Sequencher 4.8 (*1991–2007 Gene Codes Corporation*) and alignments were refined by hand with MacClade (Version 4.0, Maddison and Maddison 2000, Sinauer Associates).

### Analysis of *RCR3* flanking regions

Due to low sequence divergence at the *RCR3* ORF, it was not possible to distinguish allelic and paralogous sequences using the ORFs exclusively. Paralogs and orthologs may be distinguished by their flanking sequences since allelic sequences originate from the same locus in a genome and should possess the same (or very similar) flanking sequences. Paralogs, which are located at different positions in the genome, should have different flanking sequences. To distinguish between paralogs and orthologs, fragments of 400–2000 bp of *RCR3* flanking DNA (with a minimum of 200 bp overlap with the gene to identify the matching allele) were amplified, cloned and sequenced from individuals from the Tarapaca population of *S. peruvianu*m (individuals 7232–7241). A three-step Tail-PCR protocol with a set of random and nested *RCR3* specific primers was used (Table S1, [64]). The location of the amplified *RCR3* flanking regions in the tomato genome was assessed using BLASTn searches (http://blast.ncbi.nlm.nih.gov/Blast.cgi) and phylogenetic reconstruction (PAUP v. 4.0b10, Swofford 1999, Sinauer Associates). FLRs were assigned stringently to the different allele sequences of the *RCR3* gene, and only unambiguous pairs of alleles and FLRs were retained. Subsequently, the genomic origin of alleles with matching FLR was defined according to the BLAST search. Only *RCR3* alleles matched unambiguously to a given FLR were used for population genetic analysis.

### Population genetic analyses

The standard summary statistics including π, divergence, Tajima's *D* (*D*_T_) and Fu and Li's *D* test statistics were calculated using DnaSP v. 5.10 [65]. Sliding window analyses were also conducted using DnaSP. Phylogenetic analyses were performed using PAUP v. 4.0b10 (Swofford 1999, Sinauer Associates). The phylogenetic relationships between the sequences were determined using maximum parsimony (MP) and neighbor-joining (NJ) and these methods yielded similar topologies. To test whether gene conversion occurred between *RCR3* copies, simulations were performed assuming a recent gene duplication event with copy number variation using the coalescent simulator by Thornton (2007) [66]. We then developed an ABC method using ABCest [67] to perform the model choice procedure (between models with and without gene conversion based on the code by Beaumont *et al.* (2002) [68]) and estimate the gene conversion rate (Text S1 and S2). Additionally, we surveyed the SFS of private and shared polymorphisms for the duplicated loci [47].

To investigate whether demographic effects could explain the pattern of sequence variation at the *RCR3* locus, our results were compared to values obtained from 14 single-copy reference loci (CT066, CT093, CT099, CT114, CT143, CT148, CT166, CT179, CT189, CT198, CT208, CT251, CT268 and *sucr*), previously amplified from the same individuals of *S. peruvianum* [31], [49], [50]. A summary of their predicted gene products is found in Table 1 of [50]. Additionally, these loci were used to simulate expected neutral distributions of *D*_T_ for comparison with the observed values at the *RCR3* locus (Text S2).

### Cloning procedure and *Agrobacterium*-mediated transient expression

A total of 54 *RCR3* variants, which had been cloned into TOPO Zero Blunt for sequence analyses, were selected for functional testing. Cloning procedures of these variants were conducted according to the protocol described in [24]. Each *RCR3* variant to be functionally tested was excised from the Zero Blunt TOPO vector using the restriction enzymes XhoI and NcoI, for which restriction sites resided in the PCR primers. Excised fragments were cloned into the pFK26 vector carrying the 35S overexpression promoter. 35S::RCR3 cassettes were shuttled into the binary vector pTP05 using the restriction enzymes XbaI and SalI [24]. All clones were verified by sequencing and electroporated into *Agrobacterium tumefaciens* strain GV3101. Agroinfiltration into leaves of *N. benthamiana* plants was performed as described previously [24]. After protein expression, RCR3 is secreted into the intercellular space outside the cytoplasm membrane. To recover expressed RCR3, infiltrated *N. benthamiana* leaves were harvested 72 h post inoculation, and the apoplastic intercellular fluids (AFs) of all infiltrated leaves were isolated according to [24]. Volumes of AFs containing equal concentrations of active RCR3 were used for all further experiments. Western Blot analysis was used to confirm the expression of RCR3 using RCR3 antibodies described previously [25].

### Activity-based protein profiling and inhibition assays

Activity-based protein profiling (ABPP) using fluorescent DCG-04 [52] was used to detect RCR3 activity in the isolated AFs. 45 µl of AF were labeled with 2 µM fluorescent Bodipy-DCG-04 at pH 5.5 in the presence of 1 mM DTT for 5 h as described previously [24]. Concentrations of active RCR3 were adjusted based on the fluorescence signal. Accuracy of these adjustments was confirmed by independent ABPPs. Inhibition studies were performed by pre-incubation with 100 nM affinity-purified AVR2 [24], followed by ABPP. Proteins were separated via sodium dodecyl sulphate polyacrylamide gel electrophoresis (SDS-PAGE) and fluorescently-labeled proteins were detected by in-gel fluorescence scanning using a Typhoon 8600 scanner (GE Healthcare Life Sciences, http://www.gelifesciences.com) at ex/em 580 nm.

### HR assays

We investigated whether different RCR3 constructs could activate the hypersensitive response upon exposure to AVR2 in cultivated tomato plants (*S. lycopersicum* cv. Money Maker). For this purpose, 100 µl of AF containing equal concentrations of expressed, active RCR3 with or without 100 nM AVR2 were infiltrated into Cf-2/rcr3-3 and Cf0/RCR3 tomato leaves. Tissue collapse was monitored daily until six days post inoculation (dpi) and recorded photographically.

### Association of genotypic and observed phenotypic data

A general linear model algorithm implemented in TASSEL v. 3.0 (http://www.maizegenetics.net/) was used to evaluate correlations between phenotypic variation and sequence polymorphism at the *RCR3* locus. The genotypic data was filtered such that only mutations that occurred in frequencies greater than 25% were included. Resulting *P*-values were Bonferroni-corrected for multiple testing. A structural model of the RCR3 protease domain was created as previously described in [24] using papain (PDB code 9papA) as a template.

### RT–PCR

Seven RCR3 constructs failed to be expressed in *N. benthamiana* leaves. To confirm that the construct was designed correctly and that the agroinfiltration was successful, RNA of infiltrated leaves was isolated and RT-PCR with *RCR3*-specific primers was performed. The extraction of RNA was conducted using the Rneasy Plus Mini Kit (Qiagen) starting with 40–80 mg of plant material. cDNA-banks were created by reverse transcription using SuperScript Reverse Transcriptase (Invitrogen). RT-PCR was conducted for the *RCR3* gene and a portion of the Ribulose-bisphosphate-carboxylase-oxigenase as a RNA-extraction control (Table S1).

All sequences have been deposited on GenBank under accession numbers JQ927229–JQ927299.

## Supporting Information

Figure S1 One of 1,000 most parsimonious trees of the 3′FLR of the *RCR3* gene (indicated in black in the sketch of the *RCR3* locus). This tree was obtained by heuristic search with bootstrap support. *S. lycopersicoides* was used as outgroup.
(PDF)

Figure S2 ABC estimates of parameters for Model 2 with gene conversion (for *RCR3* ORFs). Left panel: Density of the distribution of Euclidian distances (δ) for all 100,000 simulated datasets. The blue line indicates the best 500 retained datasets after the rejection. Middle panel: Density of the posterior distribution for the gene conversion rate (*C* = 4*Nc* per nucleotide), in red is the density of the uniform prior. Dotted lines indicate the 95% credibility intervals and the mode of the distribution. Right panel: Density of the posterior distribution for the mean length of the gene conversion tract in bp, in red is the density of the uniform prior. Dotted lines indicate the 95% credibility intervals and the mode of the distribution.
(PDF)

Figure S3 Frequency spectrum of derived shared and private polymorphisms at the two *RCR3* loci and their 3′FLRs. The outgroup sequence (*S. lycopersicum*) was used to define derived polymorphisms. Shared polymorphisms occur in alleles from both *RCR3* loci. Private polymorphisms occur in only one locus. Fixed polymorphisms are fixed in one of the loci and do not occur in the other one.
(PDF)

Figure S4 One of 1,000 most parsimonious gene trees of all assigned *RCR3* alleles, obtained by heuristic search of the coding sequence (indicated in black in the structure of the gene) of the *RCR3* gene. Gaps were considered as a fifth state. Bootstrap proportions of 1,000 bootstrap replicates >500 are indicated on the branches. The *RCR3* sequence of the outgroup *S. lycopersicoides* was used to root the tree.
(PDF)

Figure S5 Distribution of neutral expectation of Tajima's *D* at the *RCR3* ORF based on 2,000 coalescent simulations. The expected neutral distributions for *Locus A* and *B* (solid and dashed lines) were obtained under a model with gene conversion. The observed values (black and grey arrows) are within the 95% confidence interval of the expected distribution (indicated by the grey area under the curves with vertical and diagonal lines, *P* = 0.125 and 0.175 for *Locus A* and *B*, respectively).
(PDF)

Figure S6 Posterior distributions of the parameters of the demographic model of the Tarapaca population with past expansion based on 14 reference loci (for *RCR3* 3′FLRs). In red is the density of the uniform prior. Dotted lines indicate the 95% credibility intervals and the mode of the distribution. Left panel: Density of the posterior distribution for population mutation rate (θ per nucleotide). Middle panel: Density of the posterior distribution for the expansion factor. Right panel: Density of the posterior distribution for the time of the expansion (in 4*N* generations).
(PDF)

Figure S7 Western Blot of all isolated AFs containing the expressed RCR3 constructs. Overexpressed RCR3 constructs were confirmed using RCR3 specific antibodies. Proteins were separated on 12% protein gels. AFs without overexpressed RCR3 were used as a negative control. AFs containing RCR3 from *S. lycopersicum* (*cv.* Rio Grande) were used as a positive control.
(PDF)

Figure S8 RT-PCR with RCR3 constructs that failed to accumulate in *N. benthamiana* AFs. RT-PCR was conducted for the *RCR3* gene and a portion of the Ribulose-bisphosphate-carboxylase-oxigenase as RNA-extraction control. PCR from genomic DNA was used to test if splicing of the *RCR3* intron had occurred. AFs not expressing any RCR3 construct were used as negative control for RNA-extraction.
(PDF)

Figure S9 Protein haplotypes of different RCR3 constructs. The protein sequence of *S. lycopersicum* (*esc*) is given in the one-letter code of amino acids and used as a reference. Only variable amino acid positions are shown. Amino acids that are identical to the *esc* sequence are indicated with dots, similar amino acids with blue, dissimilar amino acids with red, functionally relevant amino acid changes with yellow and deletions with grey. X = variant causing incompatibility with Cf2 [56], O = variant causing insensitivity to inhibition by AVR2 [24]; ^a^identical at the protein level to peru7236_A1, ^b^identical to peru7234_A1, ^c^identical to peru7234_B1 and peru7234_B2, ^d^identical to peru7233_A1, peru7238_A1 and peru7240A1, ^e^identical to peru7232_C2, ^f^identical to peru7238_A2, ^g^identical to esc_VFNTCherry, ^h^identical to peru7235_B2, peru7236_B1 and peru7241_B1.
(PDF)

Figure S10 Protease activity profiling of all RCR3 constructs. One representative result out of at least three independent replicates is shown. AFs that contained overexpressed RCR3 constructs were labeled with DCG-04 at pH 5.5. Proteins were separated on 12% protein gels. AFs without overexpressed RCR3 were used as a negative control. AFs containing RCR3 from *S. lycopersicum* (*cv.* Rio Grande) were used as a positive control.
(PDF)

Figure S11 Phenotypic evaluation of a subset of *RCR3* alleles. One representative result out of at least three independent replicates is shown. (A) Variable amino acids in the protease domain of the shown alleles: red = dissimilar amino acid, blue = similar amino acid, orange = functionally relevant amino acid. (B) Inhibition assays with AVR2. AF without overexpressed RCR3 was used as a negative control. Expression of each RCR3 construct was confirmed by protein blots using αRCR3 for detection. Despite lower concentration, chil1930_1 was less inhibited by AVR2. (C) *In planta* assays of RCR3 alleles. All active *RCR3* constructs were co-infiltrated into Cf-2/rcr3-3 and Cf0/RCR3^pim^ tomato plants with AVR2 or buffer. Necrotic lesions indicate HR. Yellow discoloration of the leave tissue indicates weak HR.
(PDF)

Figure S12 Association of SNPs along the *RCR3* locus with inhibition by AVR2 *in vitro* (A) and *in planta* (B). SNPs were correlated with the observed phenotype using a general linear model. The Y-axes on the left hand side show the *P*-values of the correlation. The Y-axes on the right hand side show the correlation coefficient. Values were corrected by the Bonferroni method. The dashed line indicates the significance threshold after Bonferroni correction (0.01). (A) Association with insensitivity to inhibition by AVR2 *in vitro*. (B) Association with inability to elicit HR after co-infiltration with AVR2 into Cf-2/rcr3-3 tomato plants.
(PDF)

Figure S13 Structural model of the RCR3 protease domain. Amino acids that are associated with insensitivity to AVR2 inhibition, the weak HR response or incompatibility between RCR3 and Cf-2 are highlighted. Left: view on the protein focused on the catalytic center. Right: view on the protein after 180° horizontal rotation.
(PDF)

Table S1 List of primers and annealing temperatures used in this study.
(PDF)

Table S2 Overview of all sampled individuals and alleles. The origin of all sampled individuals per species and accession, the number of alleles that could be detected through the performed sequencing approach and the number of functionally tested alleles is summarized. When applicable, the number of alleles assigned to *Locus A*, *B* and *C* is given. n.a. = not assigned
(PDF)

Table S3 Results of ABC estimates for gene conversion parameters at the *RCR3* ORF (Model 2 with gene conversion and variable mean tract length of gene conversion). Estimates are obtained using the best 500 simulations out of 100,000. 95% credibility intervals boundaries are shown.
(PDF)

Table S4 Summary statistics and neutrality test results calculated at the *RCR3 Locus A* and *Locus B* and their 3′FLRs in the *S. peruvianum* population Tarapaca. Both 3′FLRs were analyzed until the point of divergence between *Locus B* and *Locus A* and the *S. lycopersicum RCR3* locus (580 bp downstream of stop codon). Allele peru7241_A1 was excluded from the analysis of the 3′FLRs, because it did not span the full analyzed sequence length.
(PDF)

Table S5 Summary of all phenotypic results of the different RCR3 constructs. The origin of each construct and all phenotypic results including protein accumulation in AFs, activity-based protein profiling, inhibition by AVR2 and HR-response are shown. Constructs are named according to their species, their accession or individual number and their origin from *Locus A*, *B* or *C* in those cases for which unambiguous assignment was possible. ^a^identical at the protein level to peru7236_A1, ^b^identical to peru7234_A1, ^c^identical to peru7234_B1 and peru7234_B2, ^d^identical to peru7233_A1, peru7238_A1 and peru7240_A1, ^e^identical to peru7232_C2, ^f^identical to peru7238_A2, ^g^identical to esc_VFNTCherry, ^h^identical to peru7235_B2, peru7236_B1 and peru7241_B1. + = phenotype present, − = phenotype absent, (+) = weak response, n.t. = not tested.
(PDF)

Text S1 Evolutionary history of the *RCR3* ORFs.
(PDF)

Text S2 Evolutionary history of the *RCR3* 3′FLRs.
(PDF)

Text S3 Pseudogenized *RCR3* alleles.
(PDF)
