# Supplementary figures and images for: Balancing Selection at the Tomato RCR3 Guardee Gene Family Maintains Variation in Strength of Pathogen Defense
Source: PLoS Genet. 2012 Jul 19;8(7):e1002813. doi: 10.1371/journal.pgen.1002813 (PMC3400550; doi:10.1371/journal.pgen.1002813)

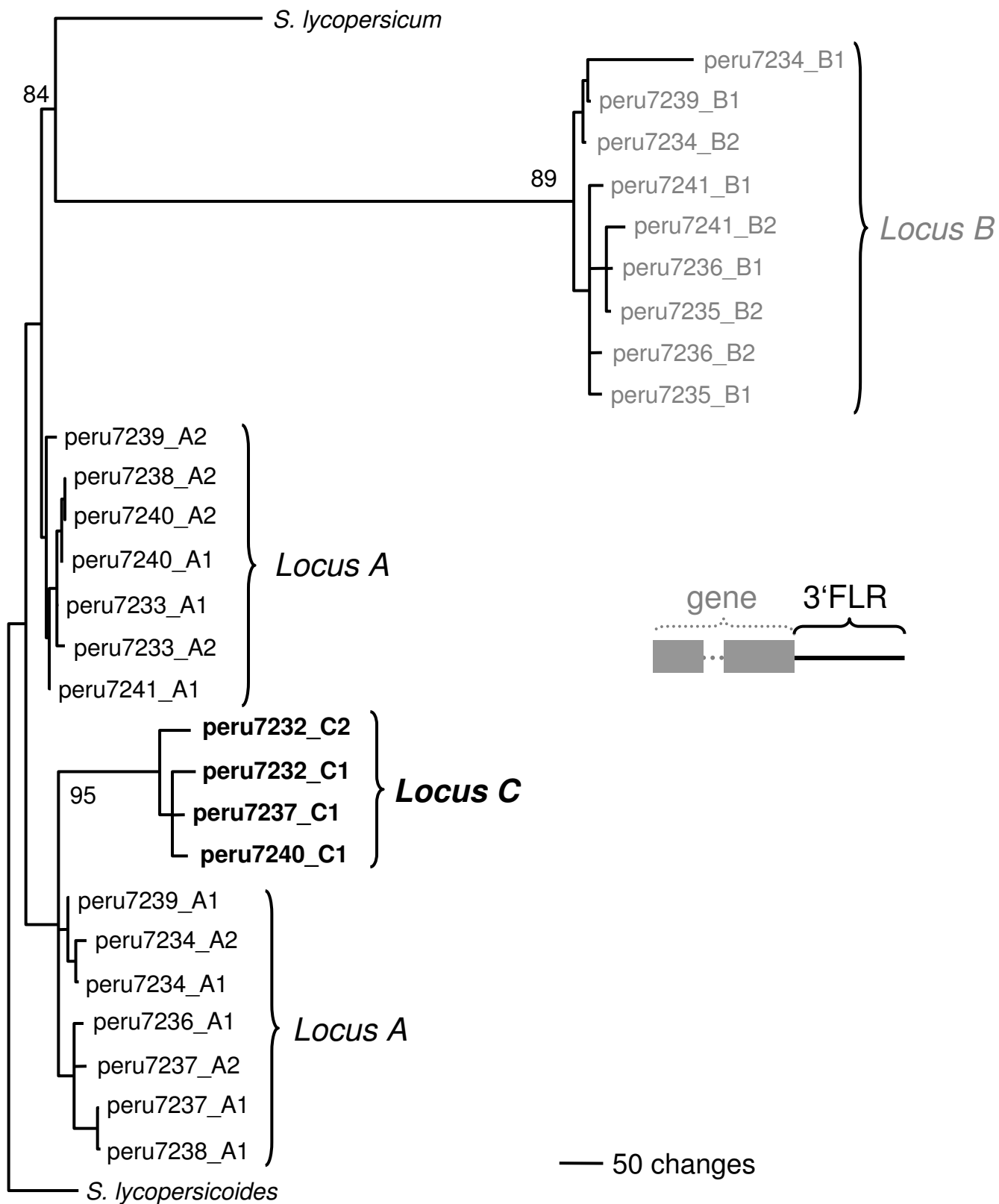

Supplement: Figure S1 — One of 1,000 most parsimonious trees of the 3′FLR of the RCR3 gene (indicated in black in the sketch of the RCR3 locus). This tree was obtained by heuristic search with bootstrap support. S. lycopersicoides was used as outgroup. (PDF) [file pgen.1002813.s001.pdf]

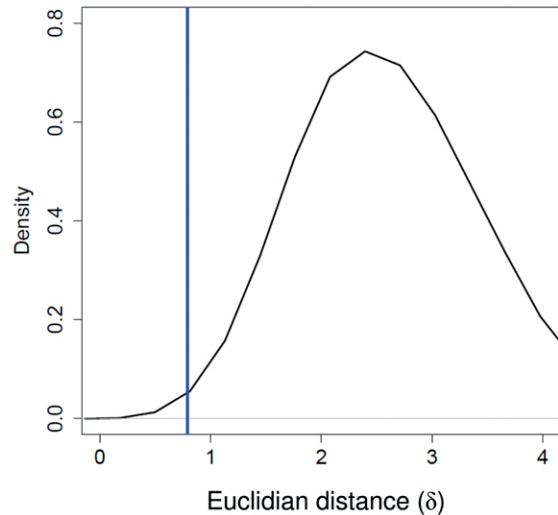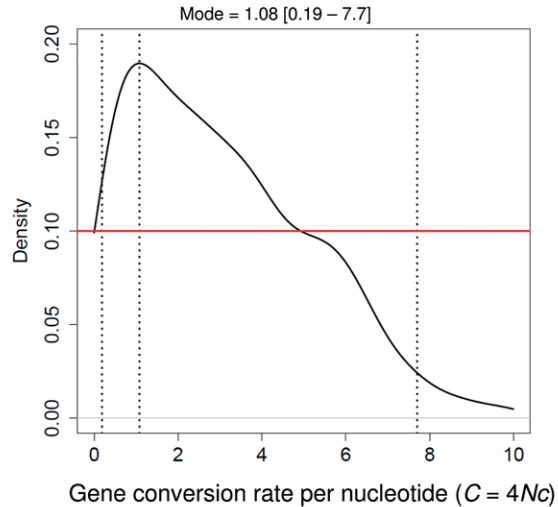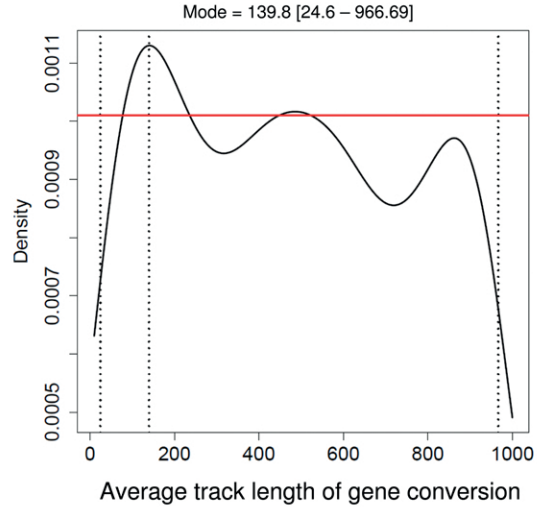

Supplement: Figure S2 — ABC estimates of parameters for Model 2 with gene conversion (for RCR3 ORFs). Left panel: Density of the distribution of Euclidian distances (δ) for all 100,000 simulated datasets. The blue line indicates the best 500 retained datasets after the rejection. Middle panel: Density of the posterior distribution for the gene conversion rate (C = 4Nc per nucleotide), in red is the density of the uniform prior. Dotted lines indicate the 95% credibility intervals and the mode of the distribution. Right panel: Density of the posterior distribution for the mean length of the gene conversion tract in bp, in red is the density of the uniform prior. Dotted lines indicate the 95% credibility intervals and the mode of the distribution. (PDF) [file pgen.1002813.s002.pdf]

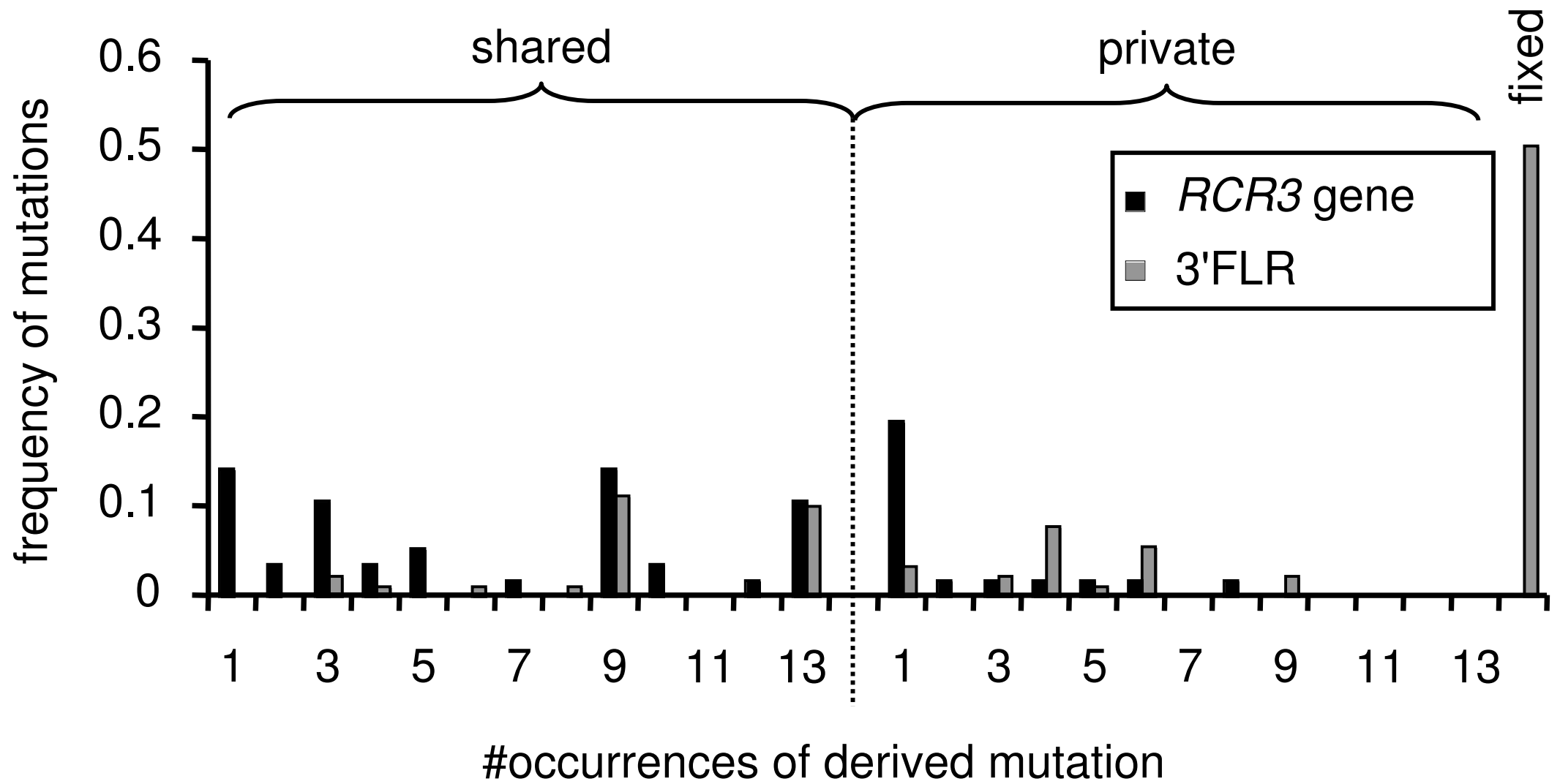

Supplement: Figure S3 — Frequency spectrum of derived shared and private polymorphisms at the two RCR3 loci and their 3′FLRs. The outgroup sequence (S. lycopersicum) was used to define derived polymorphisms. Shared polymorphisms occur in alleles from both RCR3 loci. Private polymorphisms occur in only one locus. Fixed polymorphisms are fixed in one of the loci and do not occur in the other one. (PDF) [file pgen.1002813.s003.pdf]

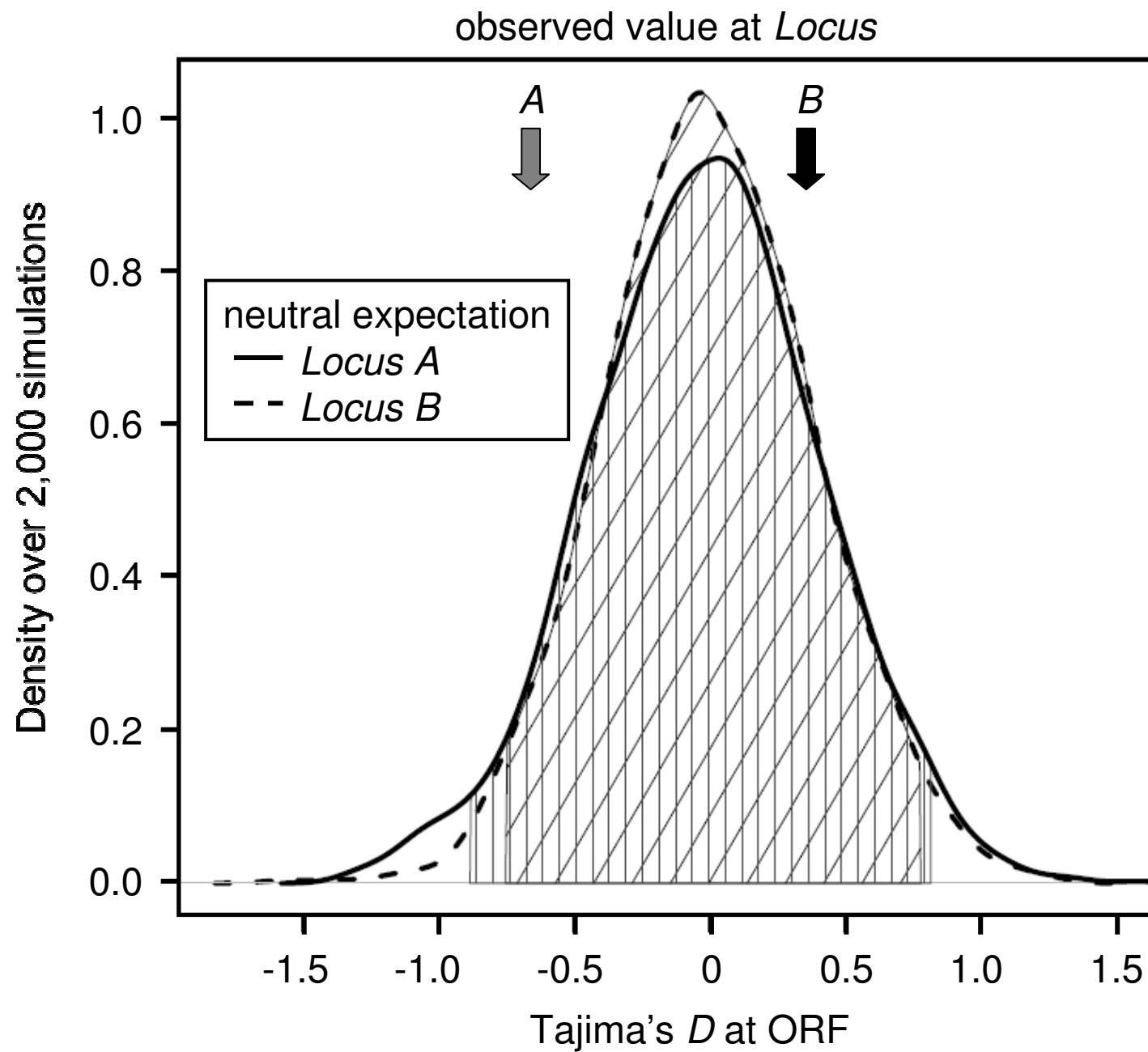

Supplement: Figure S5 — Distribution of neutral expectation of Tajima's D at the RCR3 ORF based on 2,000 coalescent simulations. The expected neutral distributions for Locus A and B (solid and dashed lines) were obtained under a model with gene conversion. The observed values (black and grey arrows) are within the 95% confidence interval of the expected distribution (indicated by the grey area under the curves with vertical and diagonal lines, P = 0.125 and 0.175 for Locus A and B, respectively). (PDF) [file pgen.1002813.s005.pdf]

Mode = 0.031 [0.025 – 0.044]

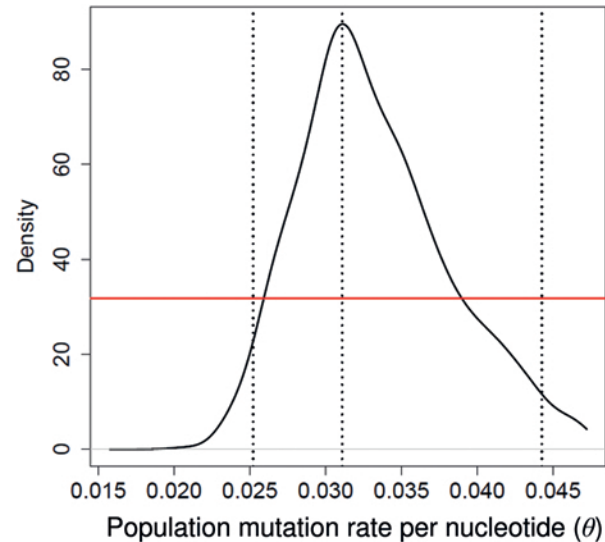

Mode = 0.405 [0.019 – 0.812]

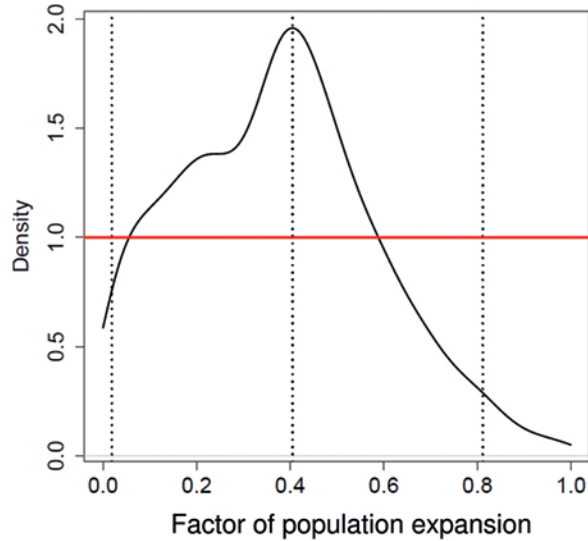

Mode = 0.479 [0.053 – 0.089]

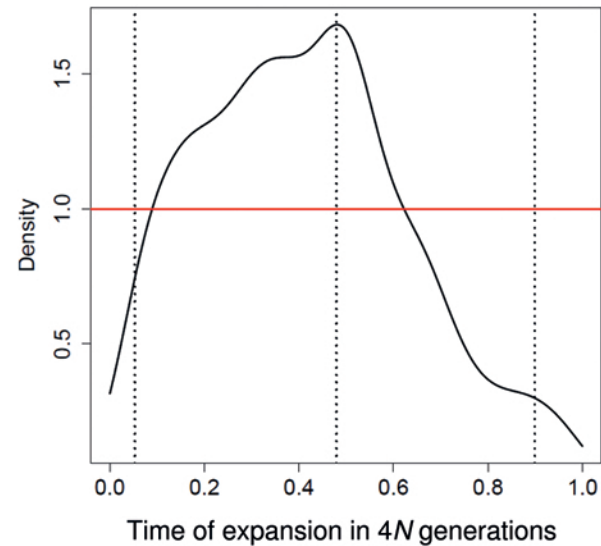

Supplement: Figure S6 — Posterior distributions of the parameters of the demographic model of the Tarapaca population with past expansion based on 14 reference loci (for RCR3 3′FLRs). In red is the density of the uniform prior. Dotted lines indicate the 95% credibility intervals and the mode of the distribution. Left panel: Density of the posterior distribution for population mutation rate (θ per nucleotide). Middle panel: Density of the posterior distribution for the expansion factor. Right panel: Density of the posterior distribution for the time of the expansion (in 4N generations). (PDF) [file pgen.1002813.s006.pdf]

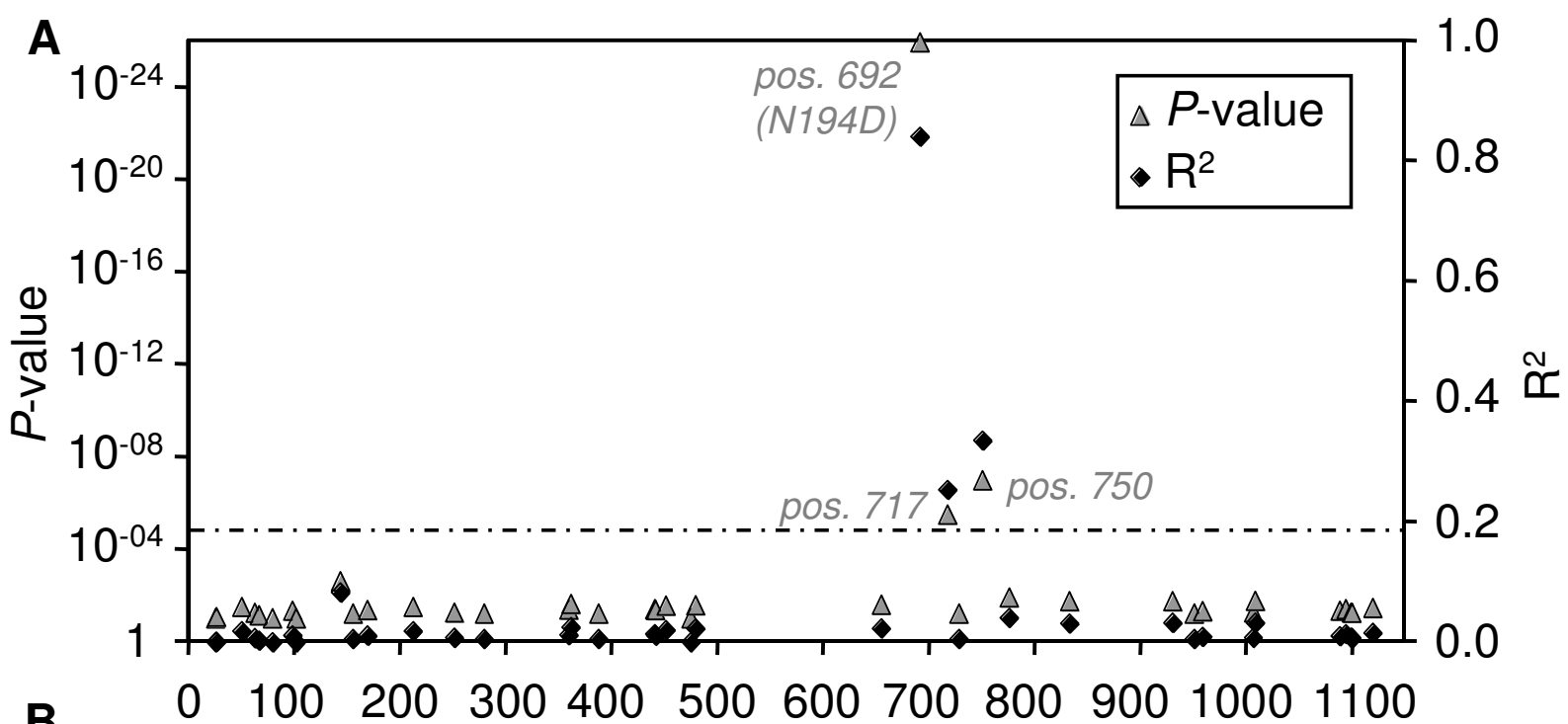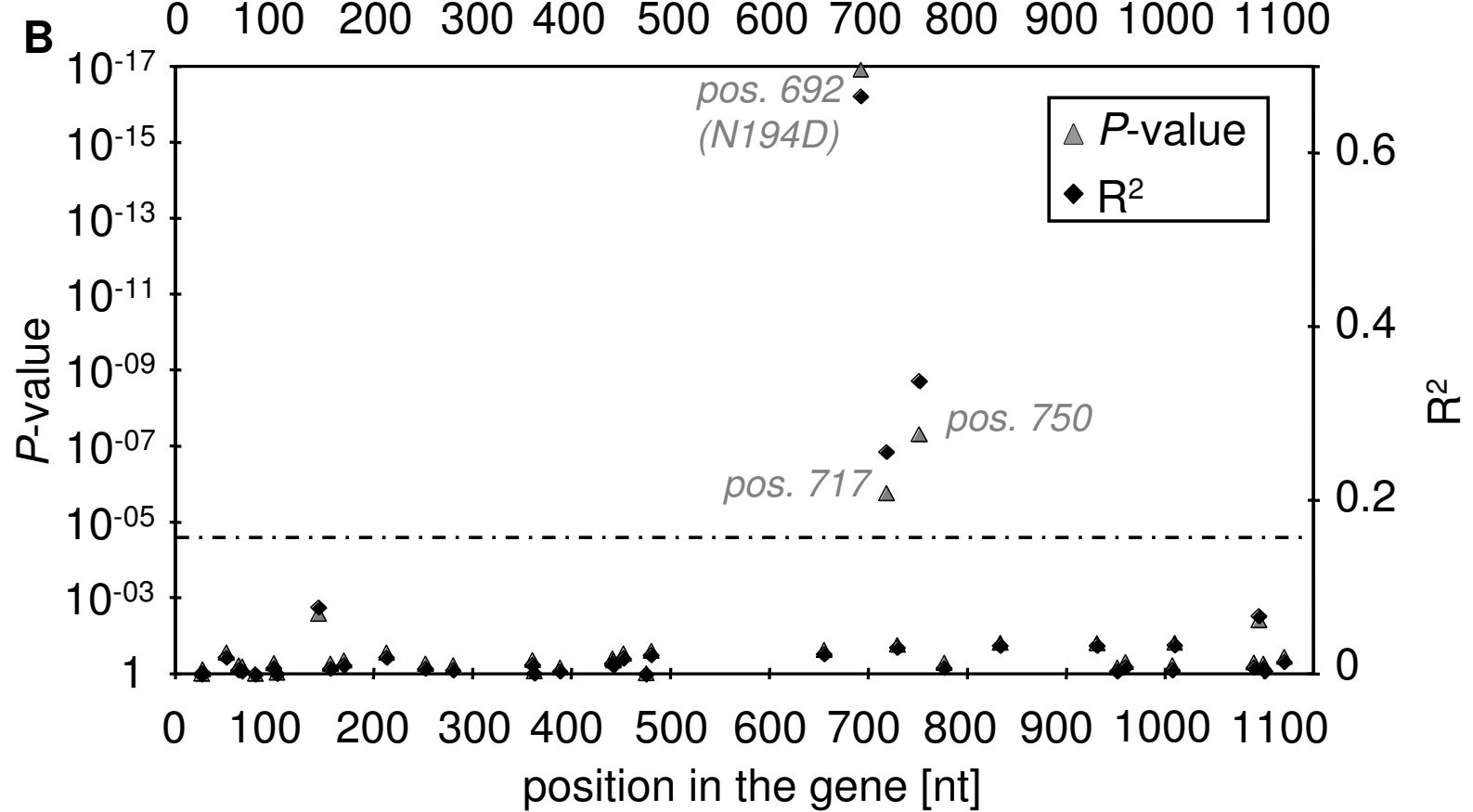

Supplement: Figure S12 — Association of SNPs along the RCR3 locus with inhibition by AVR2 in vitro (A) and in planta (B). SNPs were correlated with the observed phenotype using a general linear model. The Y-axes on the left hand side show the P-values of the correlation. The Y-axes on the right hand side show the correlation coefficient. Values were corrected by the Bonferroni method. The dashed line indicates the significance threshold after Bonferroni correction (0.01). (A) Association with insensitivity to inhibition by AVR2 in vitro. (B) Association with inability to elicit HR after co-infiltration with AVR2 into Cf-2/rcr3-3 tomato plants. (PDF) [file pgen.1002813.s012.pdf]
