# Supplementary material for: Balancing Selection at the Tomato RCR3 Guardee Gene Family Maintains Variation in Strength of Pathogen Defense
Source: PLoS Genet. 2012 Jul 19;8(7):e1002813. doi: 10.1371/journal.pgen.1002813 (PMC3400550; doi:10.1371/journal.pgen.1002813)
Supplement: Figure S4 — One of 1,000 most parsimonious gene trees of all assigned RCR3 alleles, obtained by heuristic search of the coding sequence (indicated in black in the structure of the gene) of the RCR3 gene. Gaps were considered as a fifth state. Bootstrap proportions of 1,000 bootstrap replicates >500 are indicated on the branches. The RCR3 sequence of the outgroup S. lycopersicoides was used to root the tree. (PDF) [file pgen.1002813.s004.pdf]

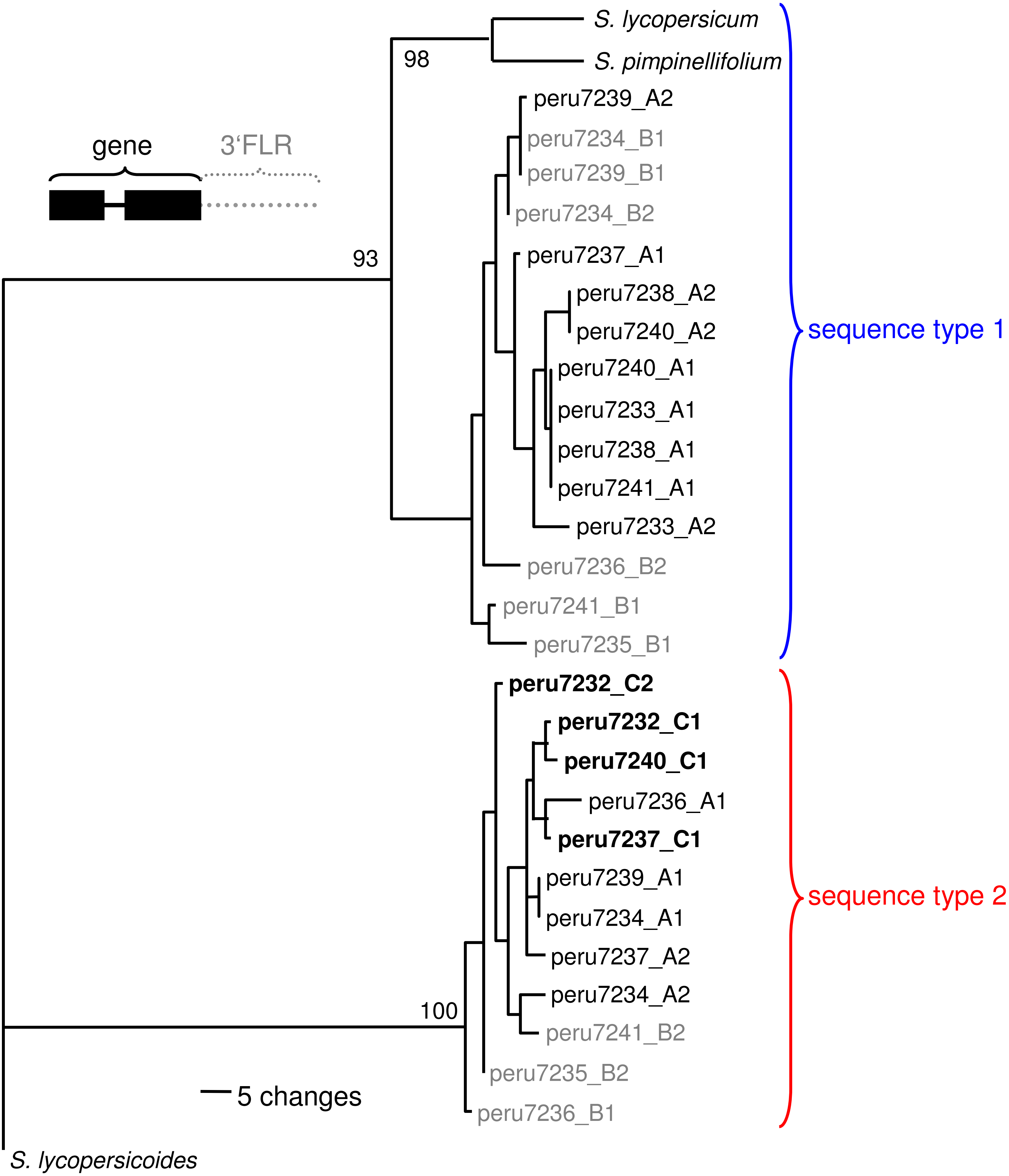
gene
3'FLR
S. lycopersicum
S. pimpinellifolium
98
93
peru7239_A2
peru7234_B1
peru7239_B1
peru7234_B2
peru7237_A1
peru7238_A2
peru7240_A2
peru7240_A1
peru7233_A1
peru7238_A1
peru7241_A1
peru7233_A2
peru7236_B2
peru7241_B1
peru7235_B1
sequence type 1
peru7232_C2
peru7232_C1
peru7240_C1
peru7236_A1
peru7237_C1
peru7239_A1
peru7234_A1
peru7237_A2
peru7234_A2
peru7241_B2
peru7235_B2
peru7236_B1
100
sequence type 2
5 changes
S. lycopersicoides
