# Supplementary material for: Balancing Selection at the Tomato RCR3 Guardee Gene Family Maintains Variation in Strength of Pathogen Defense
Source: PLoS Genet. 2012 Jul 19;8(7):e1002813. doi: 10.1371/journal.pgen.1002813 (PMC3400550; doi:10.1371/journal.pgen.1002813)
Supplement: Figure S8 — RT-PCR with RCR3 constructs that failed to accumulate in N. benthamiana AFs. RT-PCR was conducted for the RCR3 gene and a portion of the Ribulose-bisphosphate-carboxylase-oxigenase as RNA-extraction control. PCR from genomic DNA was used to test if splicing of the RCR3 intron had occurred. AFs not expressing any RCR3 construct were used as negative control for RNA-extraction. (PDF) [file pgen.1002813.s008.pdf]

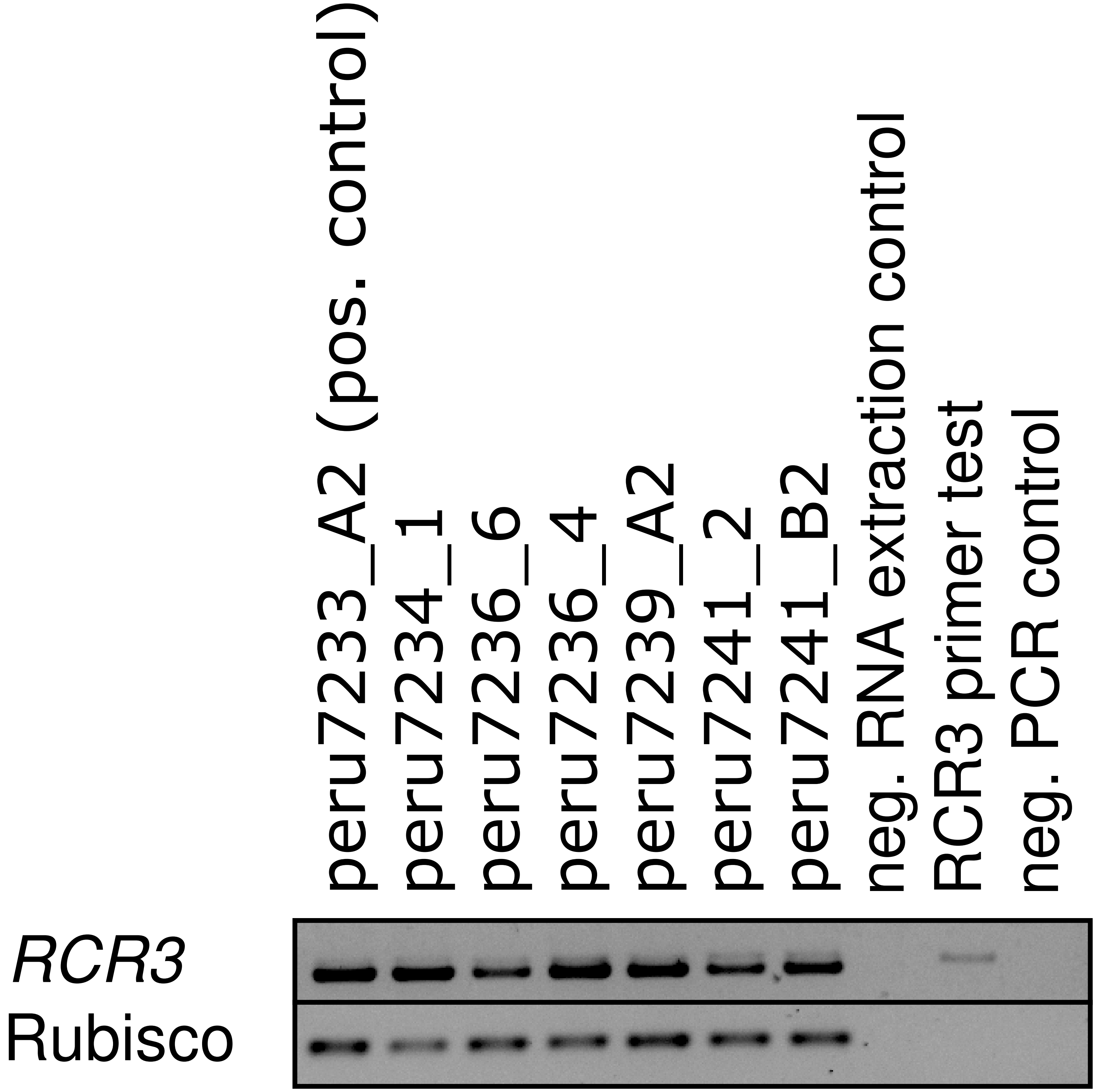

peru7233_A2 (pos. control)
peru7234_1
peru7236_6
peru7236_4
peru7239_A2
peru7241_2
peru7241_B2
neg. RNA extraction control
RCR3 primer test
neg. PCR control
RCR3
Rubisco
