# Supplementary material for: Balancing Selection at the Tomato RCR3 Guardee Gene Family Maintains Variation in Strength of Pathogen Defense
Source: PLoS Genet. 2012 Jul 19;8(7):e1002813. doi: 10.1371/journal.pgen.1002813 (PMC3400550; doi:10.1371/journal.pgen.1002813)
Supplement: Figure S10 — Protease activity profiling of all RCR3 constructs. One representative result out of at least three independent replicates is shown. AFs that contained overexpressed RCR3 constructs were labeled with DCG-04 at pH 5.5. Proteins were separated on 12% protein gels. AFs without overexpressed RCR3 were used as a negative control. AFs containing RCR3 from S. lycopersicum (cv. Rio Grande) were used as a positive control. (PDF) [file pgen.1002813.s010.pdf]

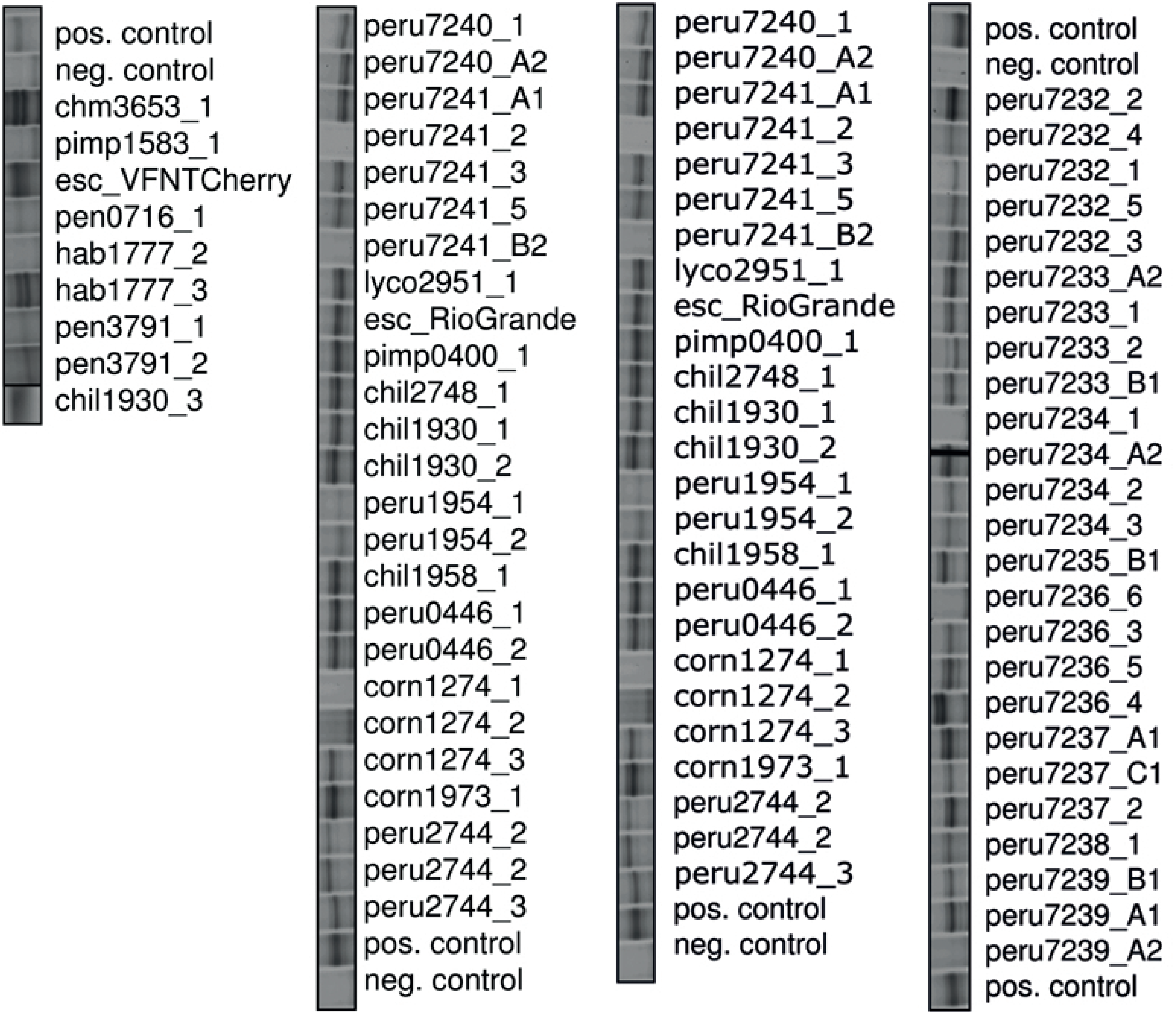
pos. control
neg. control
chm3653_1
pimp1583_1
esc_VFNTCherry
pen0716_1
hab1777_2
hab1777_3
pen3791_1
pen3791_2
chil1930_3
peru7240_1
peru7240_A2
peru7241_A1
peru7241_2
peru7241_3
peru7241_5
peru7241_B2
lyco2951_1
esc_RioGrande
pimp0400_1
chil2748_1
chil1930_1
chil1930_2
peru1954_1
peru1954_2
chil1958_1
peru0446_1
peru0446_2
corn1274_1
corn1274_2
corn1274_3
corn1973_1
peru2744_2
peru2744_2
peru2744_3
pos. control
neg. control
peru7240_1
peru7240_A2
peru7241_A1
peru7241_2
peru7241_3
peru7241_5
peru7241_B2
lyco2951_1
esc_RioGrande
pimp0400_1
chil2748_1
chil1930_1
chil1930_2
peru1954_1
peru1954_2
chil1958_1
peru0446_1
peru0446_2
corn1274_1
corn1274_2
corn1274_3
corn1973_1
peru2744_2
peru2744_2
peru2744_3
pos. control
neg. control
pos. control
neg. control
peru7232_2
peru7232_4
peru7232_1
peru7232_5
peru7232_3
peru7233_A2
peru7233_1
peru7233_2
peru7233_B1
peru7234_1
peru7234_A2
peru7234_2
peru7234_3
peru7235_B1
peru7236_6
peru7236_3
peru7236_5
peru7236_4
peru7237_A1
peru7237_C1
peru7237_2
peru7238_1
peru7239_B1
peru7239_A1
peru7239_A2
pos. control
