# Supplementary material for: Balancing Selection at the Tomato RCR3 Guardee Gene Family Maintains Variation in Strength of Pathogen Defense
Source: PLoS Genet. 2012 Jul 19;8(7):e1002813. doi: 10.1371/journal.pgen.1002813 (PMC3400550; doi:10.1371/journal.pgen.1002813)

A

	151	174	194	206	213	222	241	280	299	319	326	328	330
esc_RioGr.	R	N	N	I	R	Q	Q	N	E	Y	S	D	S
chil1930_1	R	N	D	K	S	Q	Q	S	K	F	S	N	S
peru7241_5	R	N	N	I	R	E	K	S	K	F	S	N	S
peru7241_A1	R	N	N	K	R	E	Q	S	K	F	Y	N	A
chil1930_2	Q	K	N	I	R	E	Q	S	K	F	S	N	S

B

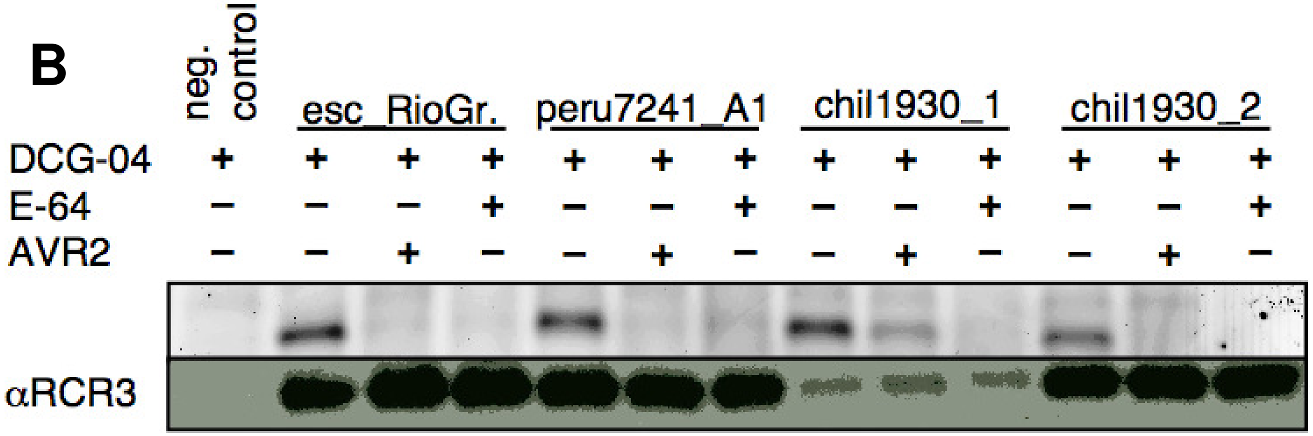

C

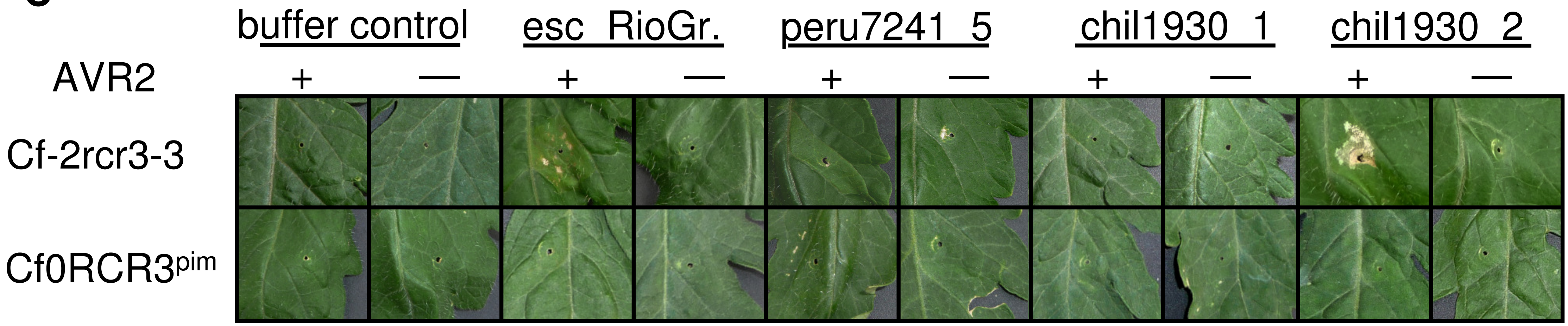

Supplement: Figure S11 — Phenotypic evaluation of a subset of RCR3 alleles. One representative result out of at least three independent replicates is shown. (A) Variable amino acids in the protease domain of the shown alleles: red = dissimilar amino acid, blue = similar amino acid, orange = functionally relevant amino acid. (B) Inhibition assays with AVR2. AF without overexpressed RCR3 was used as a negative control. Expression of each RCR3 construct was confirmed by protein blots using αRCR3 for detection. Despite lower concentration, chil1930_1 was less inhibited by AVR2. (C) In planta assays of RCR3 alleles. All active RCR3 constructs were co-infiltrated into Cf-2/rcr3-3 and Cf0/RCR3pim tomato plants with AVR2 or buffer. Necrotic lesions indicate HR. Yellow discoloration of the leave tissue indicates weak HR. (PDF) [file pgen.1002813.s011.pdf]
