# Supplementary material for: Balancing Selection at the Tomato RCR3 Guardee Gene Family Maintains Variation in Strength of Pathogen Defense
Source: PLoS Genet. 2012 Jul 19;8(7):e1002813. doi: 10.1371/journal.pgen.1002813 (PMC3400550; doi:10.1371/journal.pgen.1002813)
Supplement: Figure S13 — Structural model of the RCR3 protease domain. Amino acids that are associated with insensitivity to AVR2 inhibition, the weak HR response or incompatibility between RCR3 and Cf-2 are highlighted. Left: view on the protein focused on the catalytic center. Right: view on the protein after 180° horizontal rotation. (PDF) [file pgen.1002813.s013.pdf]

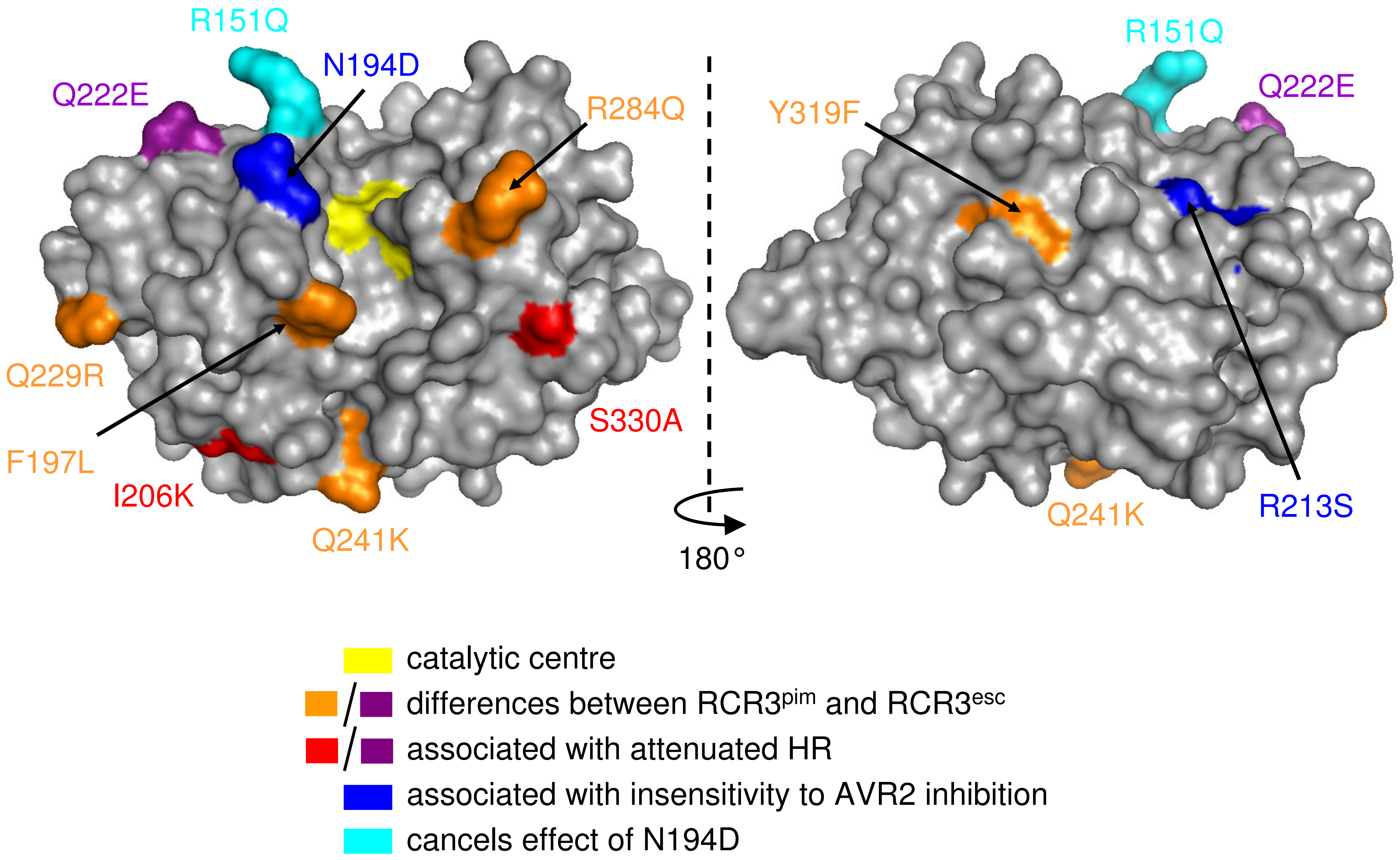
R151Q
N194D
Q222E
R284Q
Q229R
F197L
I206K
Q241K
S330A
180°
R151Q
Q222E
Y319F
Q241K
R213S
catalytic centre
differences between RCR3^{pim} and RCR3^{esc}
associated with attenuated HR
associated with insensitivity to AVR2 inhibition
cancels effect of N194D
