# Supplementary material for: Balancing Selection at the Tomato RCR3 Guardee Gene Family Maintains Variation in Strength of Pathogen Defense
Source: PLoS Genet. 2012 Jul 19;8(7):e1002813. doi: 10.1371/journal.pgen.1002813 (PMC3400550; doi:10.1371/journal.pgen.1002813)
Supplement: Table S1 — List of primers and annealing temperatures used in this study. (PDF) [file pgen.1002813.s014.pdf]

Table S1: List of primers and annealing temperatures used in this study.

Primer	Sequence 5'→ 3'	Tm [°C]	use
Primers for amplification of flanking regions			
Rcr3 3'FLR For2	GGA GGT TTT ATG ACG AAT GC	56.0	*RCR3* specific in 1st Tail-PCR step
Rcr3 3'FLR For3	CAG TAC ACA TGC AGA AGC C	57.0	*RCR3* specific in 2nd Tail-PCR step
Rcr3 3'FLR For4	GTC CAT TGG AAT AGC TGC TAG	59.0	*RCR3* specific in 3rd Tail-PCR step
Rcr3 5'FLR Rev1	CTC TCC AGT CCA AGT TAG ACG	61.0	*RCR3* specific in 1st Tail-PCR step
Rcr3 5'FLR Rev2	CTC TTG TGA AGT AAT ATC TGC	55.0	*RCR3* specific in 2nd Tail-PCR step
Rcr3 5'FLR Rev3	CTC CTT TTT CTA CTT CGT CC	56.0	*RCR3* specific in 3rd Tail-PCR step
AD1	NGT CGA SWG ANA WGA A	46.0	random priming in the flanking region
Primers for cloning procedure			
Rcr3 start	AGC TCC ATG GCT ATG AAA GTT GAT TTG ATG	68.0	amplification of *RCR3*
Rcr3 stop	AGC TCT CGA GCT ATG CTA TGT TTG GAT AAG AAG AC	73.0	amplification of *RCR3*
F401	CGT TGT AAA ACG ACG GCC AGT	61.0	forward in pFK0026
F402	CAG GAA ACA GCT ATG ACC ATG	59.0	reverse in pFK0026
F403	AGG AAG TTC ATT TCA TTT GGA GAG G	63.0	forward in 35S promotor on pFK0026
F404	CAC ATT ATA GTG ATT AGC ATG TCA C	61.0	reverse in terminator on pFK0026
r114	TAG GTT TAC CCG CCA ATA TAT CCT GTC	67.0	forward in pTP05
r115	TTC TGT CAG TTC CAA ACG TAA AAC GGC	67.0	reverse in pTP05
Primers for RT-PCR			
Rcr3RTFor1	GCC AAA ACT CTC CGT GTC TG	60.0	forward in *RCR3* for RT-PCR
Rcr3RTRev1	AGA ATC TCT TAT AAT TTT CAT AAA CC	57.0	reverse in *RCR3* for RT-PCR
Rcr3RTRev2	CAG TGA ATA ATA TTT CAT GAG ACA G	59.0	reverse in *RCR3* for RT-PCR
RubiscoRTFor1	GTT CTC GAG GAG CTT ATC AAT GG	63.0	forward in tobacco Rubisco for RT-PCR
RubiscoRTRev1	CAG GGT CCC CAT TAT CGT C	59.0	reverse in tobacco Rubisco for RT-PCR
