# Supplementary material for: Balancing Selection at the Tomato RCR3 Guardee Gene Family Maintains Variation in Strength of Pathogen Defense
Source: PLoS Genet. 2012 Jul 19;8(7):e1002813. doi: 10.1371/journal.pgen.1002813 (PMC3400550; doi:10.1371/journal.pgen.1002813)
Supplement: Table S2 — Overview of all sampled individuals and alleles. The origin of all sampled individuals per species and accession, the number of alleles that could be detected through the performed sequencing approach and the number of functionally tested alleles is summarized. When applicable, the number of alleles assigned to Locus A, B and C is given. n.a. = not assigned (PDF) [file pgen.1002813.s015.pdf]

Table S2: Overview of all sampled individuals and alleles.

species	accession no.	individuals sampled	no. of detected alleles	no. of alleles assigned to			no. of functionally tested alleles
				Locus A	***Locus B***	***Locus C***	
S. chilense	LA1930	2	4	n.a.	n.a.	n.a.	3
	LA1958	1	1	n.a.	n.a.	n.a.	1
	LA2748	1	2	n.a.	n.a.	n.a.	1
S. chmielewskii	LA3653	1	1 (homozygous)	n.a.	n.a.	n.a.	1
S. corneliomulleri	LA1274	1	4	n.a.	n.a.	n.a.	2
	LA1973	1	1	n.a.	n.a.	n.a.	1
S. habrochaites	LA1777	2	4	n.a.	n.a.	n.a.	2
S. lycopersicoides	LA2951	1	1 (homozygous)	n.a.	n.a.	n.a.	1
S. lycopersicum	cv. RioGrande	1	1 (homozygous)	1	0	0	1
	cv. VFNTCherry	1	1 (homozygous)	1	0	0	1
S. pennellii	LA0716	1	1	n.a.	n.a.	n.a.	1
	LA3791	1	2	n.a.	n.a.	n.a.	1
S. peruvianum	LA0446	1	2	n.a.	n.a.	n.a.	2
	LA1954	1	2	n.a.	n.a.	n.a.	2
	LA2744	11	43	14	9	4	33
S. pimpinellifolium	LA0400	1	1 (homozygous)	1	0	0	1
