# Supplementary material for: Balancing Selection at the Tomato RCR3 Guardee Gene Family Maintains Variation in Strength of Pathogen Defense
Source: PLoS Genet. 2012 Jul 19;8(7):e1002813. doi: 10.1371/journal.pgen.1002813 (PMC3400550; doi:10.1371/journal.pgen.1002813)
Supplement: Table S3 — Results of ABC estimates for gene conversion parameters at the RCR3 ORF (Model 2 with gene conversion and variable mean tract length of gene conversion). Estimates are obtained using the best 500 simulations out of 100,000. 95% credibility intervals boundaries are shown. (PDF) [file pgen.1002813.s016.pdf]

Table S3: Results of ABC estimates for gene conversion parameters at the *RCR3* ORF.

Parameter	Prior		Posterior		
	Lower bound	Upper bound	Mode	CI 0.025	CI 0.975
Gene conversion rate ($C = 4Nc$)	**0**	**10**	**1.08**	**0.19**	**7.70**
Mean length of gene conversion tract	**10**	**1000**	**139.80**	**24.60**	**966.69**
