# Supplementary material for: Balancing Selection at the Tomato RCR3 Guardee Gene Family Maintains Variation in Strength of Pathogen Defense
Source: PLoS Genet. 2012 Jul 19;8(7):e1002813. doi: 10.1371/journal.pgen.1002813 (PMC3400550; doi:10.1371/journal.pgen.1002813)
Supplement: Table S4 — Summary statistics and neutrality test results calculated at the RCR3 Locus A and Locus B and their 3′FLRs in the S. peruvianum population Tarapaca. Both 3′FLRs were analyzed until the point of divergence between Locus B and Locus A and the S. lycopersicum RCR3 locus (580 bp downstream of stop codon). Allele peru7241_A1 was excluded from the analysis of the 3′FLRs, because it did not span the full analyzed sequence length. (PDF) [file pgen.1002813.s017.pdf]

Table S4: Summary statistics and neutrality test results calculated at the *RCR3 Locus A* and *Locus B* and their 3'FLRs in the *S. peruvianum* population Tarapaca.

	RCR3 Locus A[#]	***RCR3 Locus A* (randomized dataset)**[+]	***RCR3 Locus B***	**3'FLR *Locus A***[#]	**3'FLR *Locus A* (randomized dataset)**[+]	**3'FLR *Locus B***	**mean at reference loci**[§]
**analyzed length**	1106	1107	1106	511	512 [511 – 516]	527	1361 [760 – 1884]
S	21	19 [13 – 21]	20	21	20 [17 – 22]	12	51 [22 – 80]
θ	0.006	0.006 [0.004 – 0.007]	0.007	0.015	0.015 [0.012 – 0.017]	0.008	0.014 [0.006 – 0.029]
π	0.005	0.005 [0.004 – 0.006]	0.008	0.017	0.017 [0.011 – 0.020]	0.011	0.013 [0.005 – 0.029]
π_s	0.008	0.008 [0.006 – 0.011]	0.021	-	-	-	0.023 [0.006 – 0.057]
π_a	0.004	0.004 [0.003 – 0.005]	0.004	-	-	-	0.0024 [0.00 – 0.007]
π_a/π_s	0.512	0.508 [0.395 – 0.730]	0.179	-	-	-	0.09 [0.00 – 0.327]
K	0.034[1]	0.034 [0.033 – 0.035]	0.035[1]	0.045[2]	0.045 [0.044 – 0.046]	0.116[2]	0.038[3] [0.012 – 0.047]
K_s	0.084[1]	0.084 [0.083 – 0.085]	0.089[1]	-	-	-	0.064[3] [0.015 – 0.096]
K_a	0.022[1]	0.022 [0.0219 – 0.0231]	0.022[1]	-	-	-	0.008[3] [0.00 – 0.024]
K_a/K_s	0.255[1]	0.255 [0.251 – 0.263]	0.233[1]	-	-	-	0.177[3] [0.00 – 0.504]
**Tajima's *D***	-0.649	-0.650 [-1.302 – 0.115]	0.362	1.278*	1.08 [-0.506 – 1.902]	1.462*	-0.386 [-1.518 – 0.250]
**Fu and Li's *D***	-0.568	-0.601 [-1.218 – 0.325]	-0.033	0.496	0.724 [-1.226 – 1.537]	1.201	-0.49 [-1.784 – 0.232]

[#] Values were calculated over all 13 functional alleles assigned to *Locus A*.

[+] The mean [minimum – maximum] value was computed from 30 resampled datasets drawing randomly ten out of 13 alleles from *RCR3 Locus A*.

[§] The mean [minimum – maximum] value was computed across a set of 14 reference loci.

[1]outgroup *S. lycopersicoides*, [2]outgroup *S. lycopersicum,* [3]outgroup *S. ochranthum*

* significantly outside the simulated distribution of Tajima's *D* in this population ($P < 0.01$) (Figure 1B)
