# Supplementary material for: Balancing Selection at the Tomato RCR3 Guardee Gene Family Maintains Variation in Strength of Pathogen Defense
Source: PLoS Genet. 2012 Jul 19;8(7):e1002813. doi: 10.1371/journal.pgen.1002813 (PMC3400550; doi:10.1371/journal.pgen.1002813)
Supplement: Table S5 — Summary of all phenotypic results of the different RCR3 constructs. The origin of each construct and all phenotypic results including protein accumulation in AFs, activity-based protein profiling, inhibition by AVR2 and HR-response are shown. Constructs are named according to their species, their accession or individual number and their origin from Locus A, B or C in those cases for which unambiguous assignment was possible. aidentical at the protein level to peru7236_A1, bidentical to peru7234_A1, cidentical to peru7234_B1 and peru7234_B2, didentical to peru7233_A1, peru7238_A1 and peru7240_A1, eidentical to peru7232_C2, fidentical to peru7238_A2, gidentical to esc_VFNTCherry, hidentical to peru7235_B2, peru7236_B1 and peru7241_B1. + = phenotype present, − = phenotype absent, (+) = weak response, n.t. = not tested. (PDF) [file pgen.1002813.s018.pdf]

Table S5: Summary of all phenotypic results of the different RCR3 constructs.

RCR3 construct*	Protein accumulation	Protease activity -AVR2	Protease activity +AVR2	HR-response -AVR2	HR-response +AVR2
esc_RioGrande	+	+	-	-	+
peru7233_2	+	+	-	-	-
chil1930_3	+	+	+	-	-
peru7232_5	+	+	+	-	-
peru7232_1	+	+	+	-	-
chil1930_1	+	+	+	-	-
peru1954_2	+	+	+	-	-
peru0446_2	+	+	+	-	-
peru7241_2[a]	-	-	n.t.	-	-
peru7233_3	+	+	-	-	(+)
peru7241_5	+	+	-	-	(+)
peru7234_2	+	+	-	-	(+)
peru2744_3	+	+	-	-	(+)
peru7232_2	+	+	-	-	(+)
peru7234_3	+	+	-	-	(+)
peru7236_3	+	+	-	-	(+)
peru7237_2	+	+	-	-	(+)
peru7235_B1	+	+	-	-	(+)
peru7239_A1[b]	+	+	-	-	(+)
peru7239_B1[c]	+	+	-	-	(+)
peru1954_1	+	+	-	-	(+)
peru7237_A1	+	+	-	-	(+)
peru7241_A1[d]	+	+	-	-	+
peru7233_1	+	+	-	-	+
peru7237_C1	+	+	-	-	+
peru7241_3	+	+	-	-	+
corn1973_1	+	+	-	-	+
chil2748_1	+	+	-	-	+
peru0446_1	+	+	-	-	+
peru2744_1[e]	+	+	-	-	+
hab1777_1	+	+	-	-	+
chil1958_1	+	+	-	-	+
peru7234_A2	+	+	-	-	+
peru7236_4	+	+	-	-	+
peru7236_5	+	+	-	-	+
peru7238_1	+	+	-	-	+
peru7232_4	+	+	-	-	+
lyco2951_1	+	+	-	-	+
peru7240_A2[f]	+	+	-	-	+
corn1274_3	+	+	-	-	+
chil1930_2	+	+	-	-	+
pimp0400_1[g]	+	+	-	-	+
hab1777_3	+	+	-	-	+
chm3653_1	+	+	-	-	+
pen0716_1	+	+	-	-	+
pen3791_2	+	+	-	-	+
peru7233_A2	+	+	-	-	+
peru7232_3	+	+	-	-	+
peru7240_1[h]	+	+	-	-	+
corn1274_1	-	-	n.t.	n.t.	n.t.
peru7241_B2	-	-	n.t.	n.t.	n.t.
peru7234_1	-	-	n.t.	n.t.	n.t.
peru7236_6	-	-	n.t.	n.t.	n.t.
peru7239_A2	-	-	n.t.	n.t.	n.t.

*Constructs are named according to their species and their accession or individual number whenever more than one individual was analyzed. Capital letters A, B and C indicate the origin from *Locus A*, *B* or *C* in those cases for which unambiguous assignment was possible.
