# Supplementary material for: Balancing Selection at the Tomato RCR3 Guardee Gene Family Maintains Variation in Strength of Pathogen Defense
Source: PLoS Genet. 2012 Jul 19;8(7):e1002813. doi: 10.1371/journal.pgen.1002813 (PMC3400550; doi:10.1371/journal.pgen.1002813)
Supplement: Text S1 — Evolutionary history of the RCR3 ORFs. (PDF) [file pgen.1002813.s019.pdf]

Text S1: Evolutionary history of the *RCR3* ORFs

Estimation of gene conversion between the *RCR3* loci through the site frequency spectrum (SFS) of shared and private polymorphisms: To investigate whether gene conversion occurs at the *RCR3* locus, we examined the SFS of shared and private polymorphisms ([1], Figure S3). This analysis revealed a substantial number of shared polymorphisms but no fixed differences between alleles of the two *RCR3* loci. Polymorphisms in the coding region, which are private to one of the loci, occur mainly in low frequency. The observed pattern is consistent with frequent gene conversion affecting the locus.

Estimation of gene conversion between the loci through ABC inference for the *RCR3* ORF: We developed an Approximate Bayesian Computation (ABC) method to estimate parameters of gene conversion occurring between the two recently duplicated *RCR3* loci. We used the coalescent simulator developed by K.R. Thornton [2] to simulate gene conversion between copy number variants in a population (program *cnvcoal* available on the K.R. Thornton webpage, http://www.molpopgen.org/software/coalescent.html). Our ABC algorithm was composed of three steps: simulation of datasets, model choice, and parameter estimation.

The simulation step consisted of simulating 100,000 datasets with identical features to the *RCR3* loci for each evolutionary scenario. The length of both loci was fixed to 1,100 bp (according to the analyzed length of the *RCR3* gene, Table S4). The population intergenic recombination rate ρ was assumed to be equal to the population mutation rate θ based on previous observations [3,4], and the distance between the two loci was fixed to 9,000 bp. The simulated sample sizes matched the observed data, and pseudogenized alleles were included for computing the summary statistics since they potentially contribute to gene conversion.

The population mutation rate ($\theta = 4N\mu$, where N is the effective population size) was chosen to vary uniformly between 8.8 and 9.8, based on the observed θ_W (identical results were obtained when θ varied between 4 and 12). The population rate of gene conversion was defined as $C = 4Nc$ where c is the rate of gene conversion per nucleotide per generation [5]. Each evolutionary scenario was defined by a set of parameters characterized by uniform prior distributions, from which we sampled to perform coalescent-based simulations (an R code is available upon request [6]). We computed summary statistics using the GSL C++ library and the libsequence C++ library as implemented in the software *summstats* [2,7]).

The model choice procedure was based on a weighted multinomial logistic regression [8] computed on the best 500 simulations (over the 100,000 performed per model) for which δ, the Euclidean distance between the observed summarized dataset and the simulated datasets, was the smallest ([9], Figure S2). Bayes factors were calculated as the ratio of the posterior probabilities for the tested models [10]. We tested three models. Model 1 assumed ancestral gene duplication without subsequent gene conversion ($4Nc = 0$). Model 2 assumed ancestral gene duplication with subsequent gene conversion, where the parameters are the mean length of gene conversion tracts (which varied uniformly between 10 and 1,100 bp) and the gene conversion rate C (which varied from 0 to 10). Model 3 assumed ancestral gene duplication with subsequent gene conversion, but had only the gene conversion rate C as a parameter. In this case, the mean length of the gene conversion tract was fixed to 395 bp which is the value obtained with *Geneconv* [11]. The following six summary statistics were used (with the value for the observed data):

- F_{ST} between the two loci (Hudson, Boos, Kaplan) = 0.03036
- $\pi_{between}$, the mean pairwise difference between the two loci = 10.604
- Number of fixed differences between loci = 0
- Number of shared polymorphisms between loci = 20

- Number of private polymorphisms in *Locus A* = 10
- Number of private polymorphisms in *Locus B* = 8

These statistics are described as good indicators of gene conversion [2]. The use of an additional four summary statistics (number of segregating sites per locus and π per locus) yielded identical results.

The model choice procedure revealed that Model 2 was clearly favored with a Bayes factor > 1,000 compared to the other two models. This demonstrates that gene conversion occurs between the two loci. We then estimated the posterior distributions (mode and 95% credibility intervals, CI) for the parameters of Model 2 by applying the locally weighted multivariate regression method [9] implemented in the ABCest program ([12], Table S3). The gene conversion rate has a mode of $C = 1.08$ (95% CI = [0.19, 7.7]; Figure S2). We conclude thus that the gene conversion rate is at least 100 times the mutation rate observed in this gene (θ_W per nucleotide = 0.0085). Note that the estimates for the mean length of gene conversion tracts are less reliable (Table S3).

Summary statistics for the *RCR3* ORF: To evaluate whether natural selection contributed to the maintenance of the distinct sequence types at the *RCR3* locus, several population genetic statistics were calculated at *Locus A* and *Locus B*. Putative pseudogenes (see below) were excluded from these analyses. To rule out demographic effects, which could interfere with the signature of natural selection acting at the *RCR3* locus, all statistics were compared to a set of 14 reference loci, which had previously been sequenced in five individuals (= ten alleles) from the same population of *S. peruvianum* [13,14,15]. To allow a clear statistical comparison between the 13 alleles assigned to *RCR3 Locus A* and the ten reference alleles we created 30 datasets by resampling randomly ten among the 13 alleles at *Locus A* and computed mean, minimum and maximum values for all summary statistics. Values of average pairwise nucleotide diversity π at the two analyzed *RCR3* loci are 0.005 (*Locus A*) and 0.008

(*Locus B*) and are therefore lower than the genome average of this population of *S. peruvianum* (0.013; Table S4). Synonymous nucleotide diversity π_s (0.008 at *Locus A* and 0.021 at *Locus B*) is also lower than the reference average π_s (0.023) in this population, while the nonsynonymous nucleotide diversity π_a (0.004 at *Locus A* and 0.004 at *Locus B*) is twice that at the reference loci (0.002). The ratio π_a to π_s is 0.512 for *Locus A* and 0.179 for *Locus B*, which is elevated compared to the average of the reference loci (0.09). Elevated π_a to π_s ratios can indicate a potential signature of balancing selection.

Coalescent simulations for the neutral scenario under gene conversion for the *RCR3* ORF: Since our preceding results demonstrated that the *RCR3* ORF is affected by frequent gene conversion, we aimed to compare population genetic statistics at the two loci to the neutral expectations under frequent gene conversion. We computed 2,000 coalescent simulations under Model 2 with gene duplication and gene conversion inferred from the ABC procedure, drawing parameter values from the 95% credibility intervals (for *C* and mean track length of gene conversion). This simulated dataset represents the neutral expectation for each of the two loci with gene conversion in a population of constant size (Figure S5). We indicate the observed values of Tajima's *D* at the two *RCR3* loci in Figure S5.

References

1. Innan H (2003) The coalescent and infinite-site model of a small multigene family. Genetics 163: 803-810.
2. Thornton KR (2007) The neutral coalescent process for recent gene duplications and copy-number variants. Genetics 177: 987-1000.
3. Städler T, Arunyawat U, Stephan W (2008) Population genetics of speciation in two closely related wild tomatoes (*Solanum* section *Lycopersicon*). Genetics 178: 339-350.

4. Stephan W, Langley CH (1998) DNA polymorphism in *Lycopersicon* and crossing-over per physical length. Genetics 150: 1585-1593.

5. Innan H (2003) A two-locus gene conversion model with selection and its application to the human *RHCE* and *RHD* genes. Proc Natl Acad Sci USA 100: 8793-8798.

6. R Development Core Team (2005) R: A language and environment for statistical computing. In: Computing RFfS, editor. Vienna, Austria.

7. Thornton K (2003) libsequence: a C++ class library for evolutionary genetic analysis. Bioinformatics 19: 2325-2327.

8. Fagundes NJR, Ray N, Beaumont M, Neuenschwander S, Salzano FM, et al. (2007) Statistical evaluation of alternative models of human evolution. Proc Natl Acad Sci USA 104: 17614-17619.

9. Beaumont MA, Zhang WY, Balding DJ (2002) Approximate Bayesian computation in population genetics. Genetics 162: 2025-2035.

10. Kass RE, Raftery AE (1995) Bayes factors. J Am Stat Assoc 90: 773-795.

11. Sawyer S (1989) Statistical tests for detecting gene conversion. Mol Biol Evol 6: 526-538.

12. Excoffier L, Estoup A, Cornuet JM (2005) Bayesian analysis of an admixture model with mutations and arbitrarily linked markers. Genetics 169: 1727-1738.

13. Baudry E, Kerdelhue C, Innan H, Stephan W (2001) Species and recombination effects on DNA variability in the tomato genus. Genetics 158: 1725-1735.

14. Roselius K, Stephan W, Städler T (2005) The relationship of nucleotide polymorphism, recombination rate and selection in wild tomato species. Genetics 171: 753-763.

15. Städler T, Roselius K, Stephan W (2005) Genealogical footprints of speciation processes in wild tomatoes: Demography and evidence for historical gene flow. Evolution 59: 1268-1279.

16. Krüger J, Thomas CM, Golstein C, Dixon MS, Smoker M, et al. (2002) A tomato cysteine protease required for Cf-2-dependent disease resistance and suppression of autonecrosis. Science 296: 744-747.

17. Shabab M, Shindo T, Gu C, Kaschani F, Pansuriya T, et al. (2008) Fungal effector protein AVR2 targets diversifying defense-related Cys proteases of tomato. Plant Cell 20: 1169-1183.
