# Supplementary material for: Balancing Selection at the Tomato RCR3 Guardee Gene Family Maintains Variation in Strength of Pathogen Defense
Source: PLoS Genet. 2012 Jul 19;8(7):e1002813. doi: 10.1371/journal.pgen.1002813 (PMC3400550; doi:10.1371/journal.pgen.1002813)
Supplement: Text S2 — Evolutionary history of the RCR3 3′FLRs. (PDF) [file pgen.1002813.s020.pdf]

Text S2: Evolutionary history of the *RCR3* 3'FLRs

Estimation of gene conversion between the *RCR3* loci through the SFS of shared and private polymorphisms: To evaluate whether gene conversion was present at the 3'FLRs we used the same approach as described for the *RCR3* ORFs (Text S1). First, we examined the SFS of shared and private polymorphisms, showing that fewer shared polymorphisms, more fixed differences, and more private polymorphisms are found between the 3'FLRs of the two loci than at the ORFs. Private polymorphisms are mainly found at intermediate to high frequencies (Figure S3). The pattern at the 3'FLRs suggests that gene conversion does not happen frequently (if at all) in the 3'FLRs.

ABC inference of gene conversion for the 3'FLRs: We also tested the occurrence of gene conversion at the 3'FLRs using an ABC procedure. Model 1 assumed ancestral gene duplication without subsequent gene conversion ($C = 0$). Model 2 assumed ancestral gene duplication with subsequent gene conversion. The parameters of Model 2 were the mean length of the gene conversion tract varying uniformly between 10 and 580 bp (the length of the 3'FLR until the breakpoint between the two genomic regions), and the gene conversion rate, C, varying from 0 to 10.

Similar summary statistics were chosen as above. The values of observed data are shown in parentheses: F_{ST} between the two 3'FLRs (0.0932), $\pi_{between}$, the mean pairwise difference between the two 3'FLRs (67.57), number of fixed differences (45), shared polymorphisms (12), private polymorphisms to 3'FLRs of *Locus A* (14) and *B* (6). The model choice procedure revealed that Model 1 is clearly favored with a Bayes factor > 1,000. This demonstrates that gene conversion does not occur in the 3'FLRs. Moreover, the poor fit of Model 1 to the observed data suggests that the two FLRs should be considered as

independent genomic regions, as indicated by the high values of $\pi_{between}$ and the number of fixed differences between loci.

Summary statistics: To investigate the evolutionary history of the 3'FLRs, several population genetic summary statistics were calculated (as in Text S1). Nucleotide diversity at the 3'FLRs is within the range of the reference loci at *Locus A* and lower at *Locus B* (Table S4). This is consistent with the scenario that *Locus A* is the ancestral *RCR3* copy, while *Locus B* is a duplicate, and has yet to accumulate as much variation in the FLR (where the gene conversion rate is low) as the ancestral locus.

ABC inference of neutral scenario for the 3'FLRs: Our aim was then to infer the best demographic model for the Tarapaca population based on the reference loci, and compare this expectation to that of the *RCR3* FLRs. Since no gene conversion occurs at the 3'FLRs, we assumed they are two independent loci in the genome. Their evolutionary history was therefore modeled based on the set of 14 reference genes (see Text S1). Two demographic models for the Tarapaca population were evaluated. In Model 1 population size was assumed to be constant. This model had a single parameter: the population mutation rate (θ). In Model 2, the population has experienced a past step-wise expansion. This model has three parameters: the present population mutation rate ($\theta = 4N\mu$), the factor of expansion (ratio of past over present population size) and the time of expansion (scaled in $4N$). In both models the population recombination rate was set equal to the mutation rate ($\rho = \theta$). For simplicity, the 14 loci were simulated as a concatenated unit of 19,053 bp using Hudon's *ms* coalescent software. We used the following observed summary statistics: total number of segregating sites (713), the average π_s per nucleotide over 14 loci (0.0231) and the average Tajima's D at synonymous sites over 14 loci (-0.3425). The model with demographic expansion (Model 2) was clearly favored (Bayes factor > 100) based on 200,000 simulated datasets (retaining the best 500; Figure S6).

Neutral simulations under population expansion for 3'FLRs: The final aim was to investigate whether the 3'FLRs exhibit signatures of natural selection (purifying, positive or balancing), *i.e.* a deviation from the demographic model inferred above. We computed 2,000 coalescent simulations under the demographic model with expansion, drawing parameter values from the 95% credibility intervals for θ, the expansion factor and time of expansion (Figure S6). This simulated dataset represented the neutral evolution expected over the genome in the Tarapaca population. We then compared the observed values of Tajima's D for the two 3'FLRs to this distribution (Figure 1B), revealing significantly elevated values indicative of balancing selection.
