# Supplementary material for: Balancing Selection at the Tomato RCR3 Guardee Gene Family Maintains Variation in Strength of Pathogen Defense
Source: PLoS Genet. 2012 Jul 19;8(7):e1002813. doi: 10.1371/journal.pgen.1002813 (PMC3400550; doi:10.1371/journal.pgen.1002813)
Supplement: Text S3 — Pseudogenized RCR3 alleles. (PDF) [file pgen.1002813.s021.pdf]

Text S3: Pseudogenized *RCR3* alleles

To evaluate functional differences between sequence types at the amino acid level, we overexpressed 54 naturally occurring *RCR3* alleles in *N. benthamiana* and tested them for their sensitivity to inhibition by the fungal protease inhibitor AVR2 and for their ability to activate HR *in planta* when co-infiltrated with AVR2 into *rcr3*-mutant tomato plants. After isolation of apoplastic fluids (AFs) from infiltrated *N. benthamiana* leaves, expression of the *RCR3* alleles was detected through Western blotting using RCR3 specific antibodies. Of the total number of tested alleles, 47 were detected in AFs by Western blotting (Figure S7, Table S5). The remaining seven *RCR3* alleles (peru7234_1, peru7236_A1, peru7236_6, peru7239_A2, peru7241_2, peru7241_B2 and corn1274_1) did not accumulate in multiple expression assays. To confirm that these constructs were designed correctly and that the agroinfiltration was successful, RNA of infiltrated leaves was isolated and RT-PCR with *RCR3*-specific primers was performed. The extraction of RNA was conducted using the Rneasy Plus Mini Kit (Qiagen) starting with 40-80 mg of plant material. cDNA-banks were created using SuperScript^{TM} Reverse Transcriptase (Invitrogen). RT-PCR was conducted for the *RCR3* gene and a portion of the Ribulose-bisphosphate-carboxylase-oxigenase as RNA-extraction control (Table S1). The results of the RT-PCR confirm the accumulation of the *RCR3* mRNA indicating that there were no problems with the constructs or the agroinfiltration process (Figure S8). In all of these cases in which no protein accumulated, the candidate (putatively deleterious) substitutions could be identified (Table S5, Figure S9). Five of these *RCR3* alleles that failed to accumulate have frame shift mutations, which lead to premature stop codons, potentially explaining their protein instability. The remaining two alleles that failed to accumulate each carry a single mutation, which distinguishes them from expressed alleles. These mutations likely interfere with protein stability or trafficking of the protein into the apoplast. Since these seven alleles appear to be pseudogenes, they were

excluded from the population genetic analyses for the *RCR3* ORF, but included in the gene conversion analysis.
